# Two-Layer Ultra-Wideband Localization: Scalability for Dense Wearable Motion Capture

**DOI:** 10.3390/s26185738

**Published:** 2026-09-09

**Authors:** Dominik Müller, Michael Sonnberger, Jorge F. Schmidt

**Affiliations:** Wireless and IoT, Digital Factory Vorarlberg, 6850 Dornbirn, Austria; michael.sonnberger@vactory.at (M.S.); jorge.schmidt@vactory.at (J.F.S.)

**Keywords:** ultra-wideband (UWB), wearable localization, scalability, stochastic geometry, spatial reuse, proximity-aware coordination

## Abstract

A recurrent challenge in scaling ultra-wideband (UWB) motion-capture systems is interference management when many ranging transactions coexist in time and space. To address this, we study a two-layer localization architecture that separates field-level player localization from local on-body pose tracking, allowing the two tasks to operate with different communication regimes and spatial-reuse policies. A stochastic-geometry framework is used to map sport-dependent parameters, including player density, field size, tag count, anchor count, update rates, and ranging airtime, to reliability and update-rate tradeoffs. The analytical model is parameterized using controlled experiments that characterize ranging success under temporal overlap, player distance, and variable-delay scheduling. These measurements inform the design of a proximity-aware local coordination strategy. We apply our proposed approach to soccer, volleyball, and ice hockey as representative use cases. Our results show that proximity-aware coordination can provide a scalable and lightweight interference management mechanism. Coordination is activated only where local player clustering creates strong interference, while spatially separated players continue to share resources without coordination. For the highest-density scenario tested, this increases the local-layer ranging success from below 50% without coordination to over 80% in four- and eight-player congestion clusters, while avoiding network-wide coordination overhead.

## 1. Introduction

Wearable localization is increasingly used in human motion analysis, with sport-performance optimization emerging as a challenging application. Ultra-wideband (UWB) is particularly suitable for these applications because it enables accurate ranging in indoor and body-centric environments [1,2,3,4,5]. However, as deployments become denser, scalability becomes increasingly constrained by the complexity of managing the interference among many double-sided two-way ranging (DS-TWR) transactions coexisting in time and space.

As the number of nodes increases, repeated ranging exchanges and contention reduce the update rates that can be attained when a target reliability must be ensured. Existing approaches address this issue through scheduling, protocol design, or platform-level optimizations [6,7,8,9]. These solutions are typically studied in single-layer localization networks, where all ranging interactions are treated as part of the same access problem. This view is limiting for wearable motion-capture systems, where different localization tasks operate at different spatial scales.

A key observation is that sport-oriented wearable localization naturally separates into two layers. Field-level player localization requires infrastructure coverage and moderate refresh rates, typically with only one globally visible tag per player. In contrast, on-body pose estimation requires several body-worn tags and higher-rate ranging over much shorter distances, as the relevant links are confined to the body of the same player. This creates two different interference problems. At the global layer, all player tags may contribute to the field-wide interference environment. At the local layer, interference is mainly relevant between players that are physically close, because spatially separated players can reuse the same radio resources with limited mutual impact.

**Figure 1:**
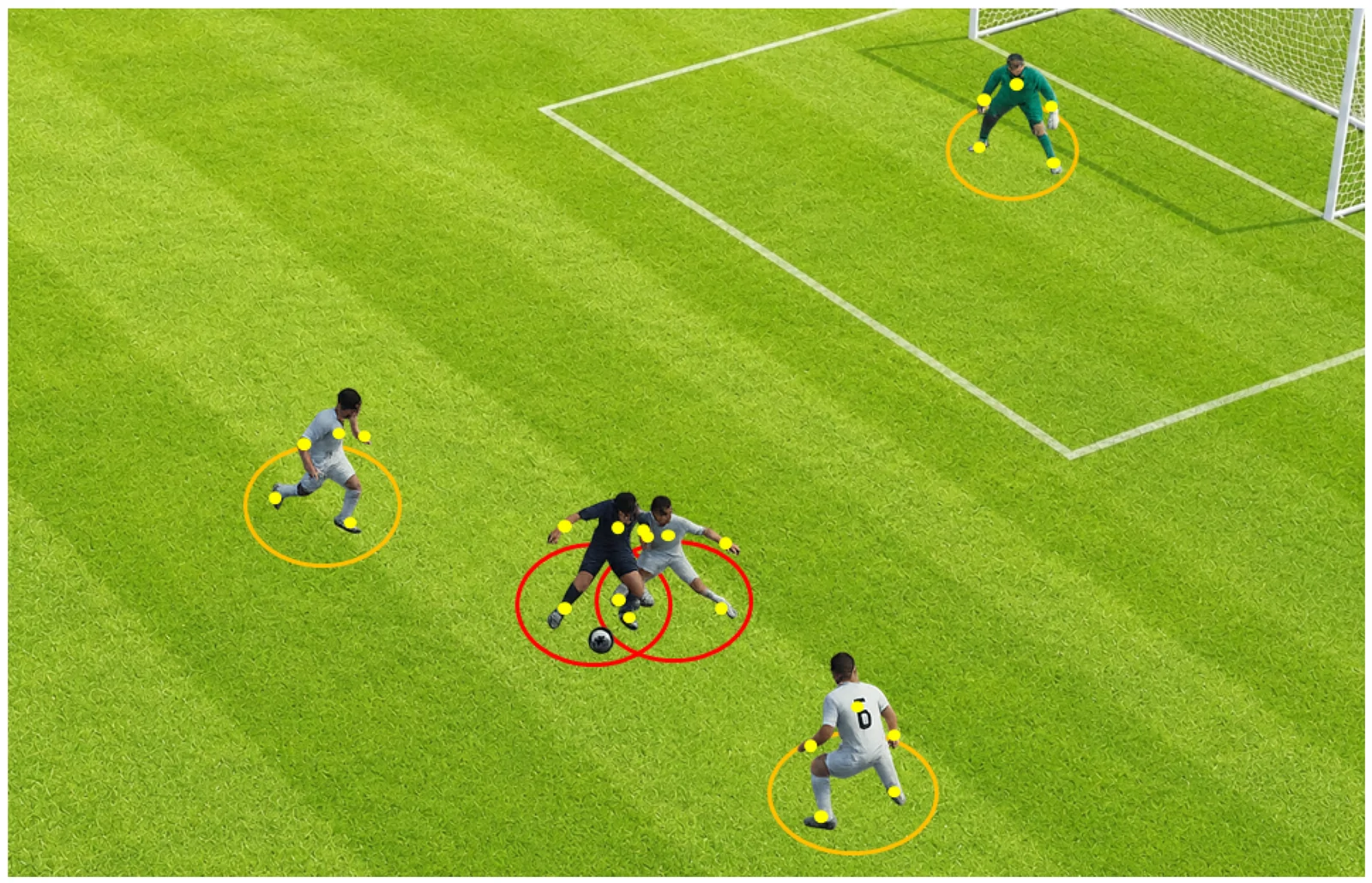
Local-layer interference scenario. Body-worn tags (yellow dots) perform on-body ranging for each player. Spatially separated players (orange circles) can reuse resources without coordination, while nearby players (red circles) would require coordination for successful pose tracking. Global-layer interference is not shown to avoid clutter.

Figure 1 illustrates the local-layer interference scenario. Each player wears several tags used for on-body ranging. Players that are sufficiently separated can operate their local (i.e., on-body) ranging networks independently, while nearby players create overlapping local interference regions and may require temporary coordination. Treating both layers as one flat ranging network would ignore this spatial structure, leading either to excessive interference under uncoordinated access or to overly conservative network-wide scheduling and higher management overhead. A two-layer architecture can instead enable spatial reuse in the local layer while reserving coordination for the cases where player proximity makes it necessary.

This paper studies such a two-layer UWB localization architecture that separates global player localization from local on-body pose tracking. We use stochastic geometry to map sport-specific and system-level parameters, including player density, field size, tag count, anchor count, update rates, and ranging airtime, to an interference scenario from which insights into reliability and its tradeoff with update rate can be extracted. The resulting analytical framework is applied to soccer, volleyball, and ice hockey as representative use cases. Controlled experiments are used to characterize the coexistence mechanisms that underlie the model, in particular temporal-overlap sensitivity, distance-dependent interference, and variable-delay scheduling. These experiments bridge the gap between approximations in our model and real implementations.

Our main contributions can be summarized as the following:

- A two-layer UWB localization architecture that separates global player localization from local on-body pose tracking, enabling lightweight interference management and better scalability.
- A stochastic-geometry framework that maps sport and system parameters to system design tradeoffs and assesses the associated operating regimes.
- A proximity-aware coordination strategy for local congestion regions that adapts spatial reuse to sport-specific clustering, simplifying network-wide coordination.
- Experimental validation of the proposed architecture, identifying vulnerable overlap regions and providing a practical evaluation of spatial reuse.

The remainder of the paper is organized as follows. Section 2 introduces the two-layer architecture and sport scenarios considered throughout the paper. Section 3 describes the experimental platform used to parameterize the analytical model. Section 4 and Section 5 develop the stochastic-geometry network model and analyze scalability tradeoffs. Section 6 introduces the proposed proximity-aware coordination and validates it experimentally, while Section 7 examines its sensitivity and the calibration of the proximity threshold. Section 8 discusses the overall design implications, Section 9 positions the work with respect to prior research, and Section 10 concludes the paper.

## 2. System Architecture and Problem Statement

**Figure 2:**
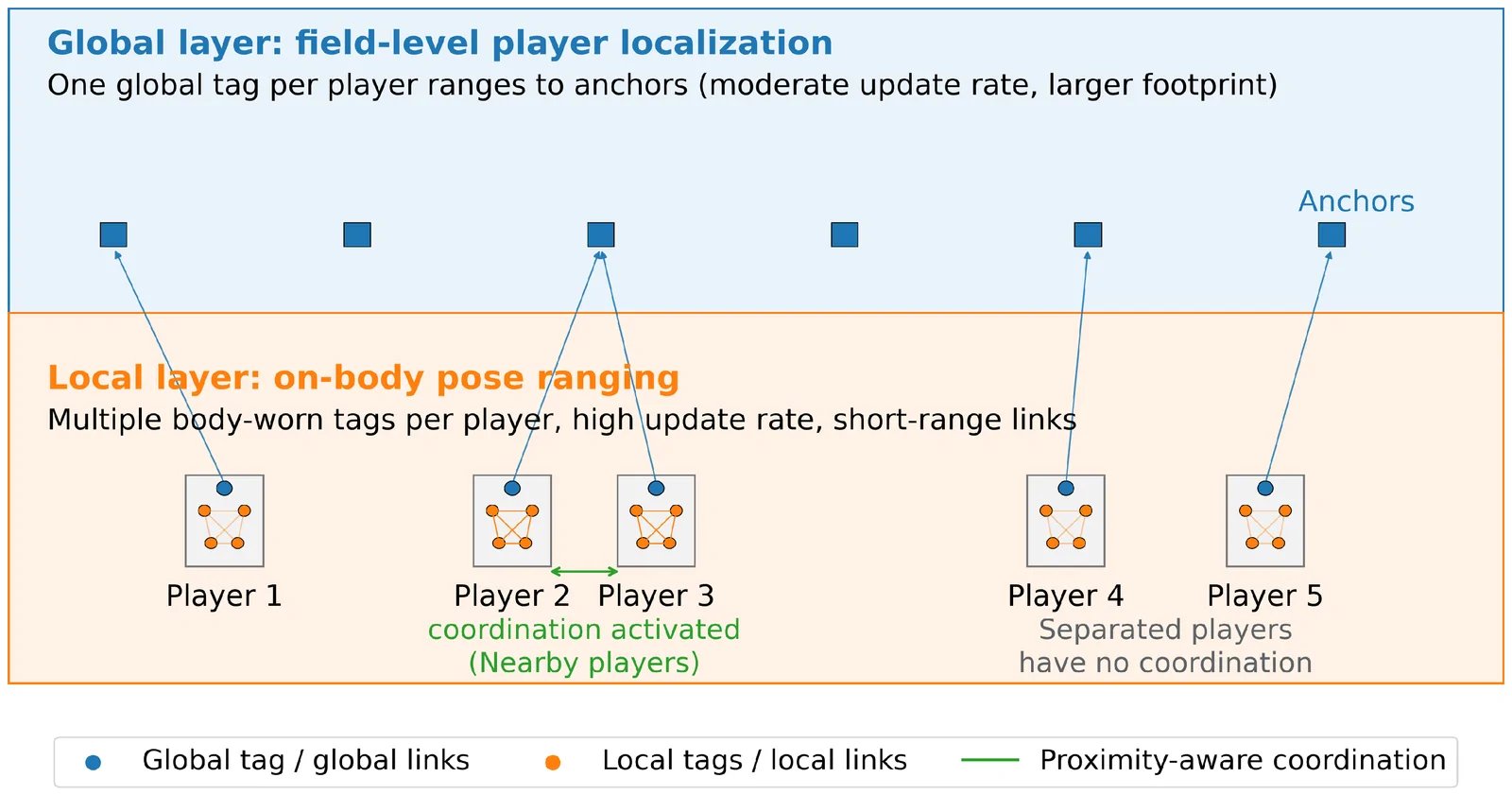
Two-layer UWB localization architecture and corresponding abstraction for the analytical model. This separation maps naturally to the model parameters: player density, anchor count, body-worn tag count, global and local update rates, and global and local ranging airtimes.

We consider a wearable localization system in which multiple players, each wearing several UWB sensors, are simultaneously tracked within a shared field. Localization is separated into two communication layers, as illustrated in Figure 2, reflecting the different spatial scales and update-rate requirements of player tracking and pose estimation. The global layer provides field-level player localization: one globally visible tag per player ranges to infrastructure anchors in order to estimate player position, requiring coverage over the full field. In contrast, the local layer supports player pose tracking through short-distance ranging among the tags worn by the same player. Although this requires several tags per player and higher local ranging activity, the relevant links are mostly confined to the body. Hence, the local layer can operate with lower transmit power and a smaller interference footprint than the global layer.

The smaller interference footprint of the local layer can be harnessed to improve system scalability. The global layer scales primarily with the number of players and anchors, whereas the local layer scales with the number of body-worn tags per player but remains spatially confined. This creates the spatial-reuse opportunity that is the focus of this paper: radio resources can be reused independently across sufficiently separated players, while additional coordination is only required for nearby players.

To make the discussion concrete, we consider three representative sport scenarios: soccer, volleyball, and ice hockey, whose deployment characteristics are summarized in Table 1, following FIFA, FIVB, and IIHF regulations, respectively. They differ substantially in field size, the number of players (and hence player density), and expected motion dynamics. Soccer represents a large-area, low-density deployment, where accurate player location tracking with full field coverage is the dominant challenge. Clustering of players requiring local coordination is limited. Volleyball represents a smaller-area, high-density deployment, where short inter-player distances and fast pose changes make local-layer coordination critical. Finally, ice hockey represents a moderate-density deployment, where rapid skating motion and the associated high pose-update rate constitute the main challenge.

These example sports illustrate how the relevant design parameters cannot be chosen independently or freely. The number of players and the deployment area are imposed by the sport, while the required global and local update rates depend on the motion dynamics to be captured. The number of body-worn sensors depends on the desired pose resolution. The following sections formalize these constraints and analyze how the two-layer architecture scales as these parameters vary.

For ease of reference, Table 2 summarizes the main system-level parameters used throughout the paper. These connect the architecture in Figure 2 and the sport scenarios in Table 1 to the stochastic-geometry model introduced in Section 4. The number of players $N_{P}$, the field area $A_{\mathrm{field}}$, and the sport-dependent update-rate requirements $f_{g}$ and $f_{l}$ define the deployment load. The number of anchors $N_{A}$ and body-worn tags $N_{T}$ define the global and local ranging demand, respectively, while the airtimes $\tau_{g}$ and $\tau_{l}$ and transmit powers $P_{g}$ and $P_{l}$ capture the main implementation- and PHY-dependent design choices.

## 3. Experimental Setup and Scenario Characterization

The stochastic-geometry model in the following sections requires hardware-dependent parameter values that cannot be set arbitrarily, because the results are sensitive to their values. In particular, the DS-TWR transaction airtime and the transmit-power settings determine how the local and global layers operate. This section therefore describes the experimental platform, provides specific values for $\tau_{l}$, $\tau_{g}$, $P_{l}$, and $P_{g}$, and explains how these are used in the analytical framework.

All experiments were conducted using DWM3001CDK development boards (Qorvo, Inc., Greensboro, NC, USA) based on the Qorvo DW3110 UWB transceiver (Qorvo, Inc., Greensboro, NC, USA) [11]. The UWB PHY was configured for IEEE 802.15.4z [12] HRP-UWB on channel 5, with center frequency 6489.6 MHz, 499.2 MHz bandwidth, 64 MHz BPRF, and a 6.8 Mbit/s data rate. The parameters varied are the preamble length and the DW3110 transmit-power configuration. Each node runs custom firmware implementing DS-TWR with configurable timeouts and a suitable local and global update schedule.

### 3.1. Measurement Setup and Data Collection

**Figure 3:**
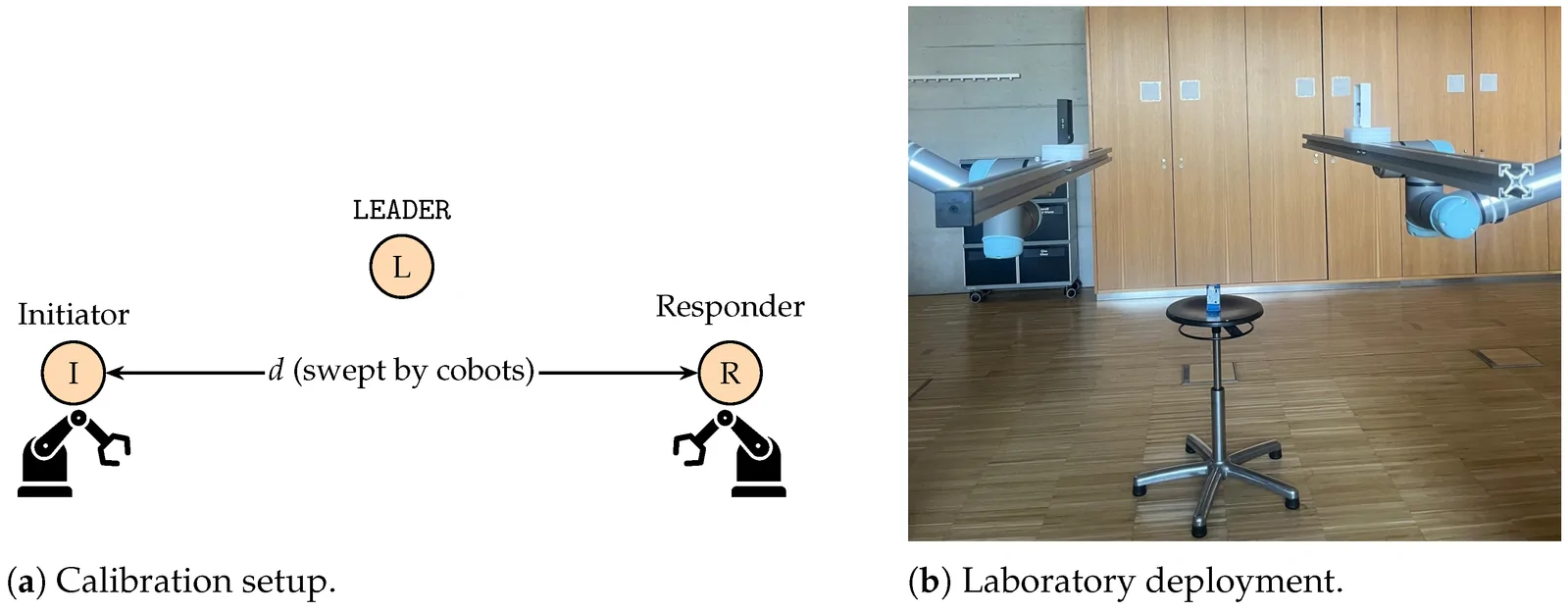
Single-network calibration setup (**a**) and laboratory deployment (**b**). The two ranging nodes are mounted on opposing cobot rails, and their separation *d* is swept to measure distance-dependent ranging success under different preamble and transmit-power settings.

The calibration setup is shown in Figure 3. It consists of a LEADER node and two ranging nodes mounted on opposing rails held by cobots. The left node acts as the DS-TWR initiator and the one on the right acts as the responder. Both operate on the same personal area network (PAN). The LEADER periodically triggers the ranging transaction, collects condensed ranging reports and timing diagnostics, and forwards the data to a host PC for post-processing in Python 3.11 (Python Software Foundation, Wilmington, DE, USA). The separation *d* between initiator and responder is varied using the available cobots for better reproducibility.

This setup provides a controlled way to measure the DS-TWR airtime associated with different preamble length configurations. It also characterizes the ranging success probability as a function of distance, preamble length, and transmit power, needed to determine which PHY settings are used for the local and global layers.

### 3.2. Ranging Airtime and Operating-Point Selection

A DS-TWR transaction consists of three frames: POLL, RESP, and FINAL. The total ranging airtime is therefore the time required to exchange the three frames and depends strongly on preamble length. Direct timing measurements on the platform give the ranging airtimes τ for preamble lengths of 32 and 1024 symbols reported in Equation (1):

(1) τ(32sym)=1339μs,τ(1024sym)=4695μs.

Thus, moving from a 32-symbol to a 1024-symbol preamble increases the DS-TWR airtime by a factor of approximately 3.5, as follows directly from Equation (1). For the local layer, where many pairwise ranges must be updated at a high rate, short preambles are preferable provided that the required link distance can be reached with the target ranging reliability.

Table 3 reports the measured DS-TWR success probability for short-range links. Each operating point aggregates 5000 ranging exchanges. At the baseline local-power setting $P_{l}$ (−32 dB relative to maximum output), the 32-symbol configuration is reliable up to about 60 cm but degrades sharply at 70 cm. Increasing the preamble length to 1024 symbols improves the link margin, but at the cost of a 3.5-fold increase in airtime. Alternatively, raising the transmit power to $P_{l}^{+}$ (−29 dB) preserves the short airtime and gives 97% success at 70 cm and 90% at 80 cm.

For the local layer, the selected operating point is therefore the 32-symbol preamble with the elevated local-power configuration $P_{l}^{+}$. This preserves the short ranging airtime given by Equation (2),

(2) $$\tau_{l}=1339\;\mu s,$$

while supporting the intended body-scale ranging distances. In the analytical model, this operating point defines the local-layer airtime and transmit power. For notational simplicity, the selected local-layer power is denoted by $P_{l}$ in the following sections.

The global layer has a different requirement: player tags must range to infrastructure anchors over substantially larger distances, and the number of global tags is only one per player. Longer airtime is therefore acceptable if it provides the required link margin. To characterize this regime, Table 4 reports measurements at a 10 dB elevated transmit-power configuration over distances from 1 to 20 m. The measurements are averaged over 5000 DS-TWR exchanges.

The long-range measurements show that transmit-power increase alone is insufficient to support the global-layer distance scale with a short preamble. Both preamble lengths are reliable up to 5 m, but at 10–15 m the 32-symbol configuration collapses to 0–6%, while the 1024-symbol configuration remains at 97–99%. The global layer is therefore represented in the model by the long-preamble operating point given by Equation (3),

(3) $$\tau_{g}=4695\;\mu s,$$

together with an elevated transmit-power setting denoted by $P_{g}$, 10 dB higher than $P_{l}$.

The calibrated operating points used in the analytical model are thus a short-airtime local layer with $\tau_{l}=1339\;\mu$s and selected local transmit power $P_{l}$, and a longer-range global layer with $\tau_{g}=4695\;\mu$s and transmit power $P_{g}$. These values are used in the activity probabilities and interference terms of the stochastic-geometry model in Section 4 and subsequent sections.

## 4. Stochastic-Geometry Network Model

Using the characterization from Section 3, we now turn to the stochastic-geometry model used to analyze the scalability of the two-layer architecture. We first define the spatial distribution of players and body-worn tags, then translate update rates and ranging airtimes into global and local activity probabilities, and finally decompose the resulting interference in order to derive ranging success probabilities and expressions for the main physical interference mechanisms.

*Spatial model:* Player locations are modeled as a homogeneous Poisson point process (PPP) $\Phi_{p}\subset R^{2}$ with density $\lambda_{p}$, representing the spatial density of players. Although the actual sport field is finite and contains a deterministic number of players, the PPP model provides an analytically tractable approximation for spatial snapshots of player configurations. For a field of area $A_{\mathrm{field}}$ containing $N_{P}$ players, the player density is given by Equation (4),

(4) $$\lambda_{p}=\frac{N_{P}}{A_{\mathrm{field}}},$$

which relates $\lambda_{p}$ to finite player numbers.

Each player forms a wearable sensor cluster. The cluster center is given by the player location $x\in \Phi_{p}$, and each cluster contains $N_{T}$ body-worn tags in total. In our notation, $N_{T}$ includes the globally tracked tag; hence, the number of purely local tags is $N_{T}-1$. The relative displacement of tag *i* with respect to the player center is denoted by $U_{x,i}$, with density $f_{U}\left(u\right)$. Unless otherwise stated, the displacements are assumed independent and identically distributed for all players, with $U_{i}∼N\left(0,\sigma^{2}I\right)$, resulting in a Thomas-type cluster process [13,14]. The number of tags per cluster is deterministic and equal to $N_{T}$, reflecting the actual system design.

The set of body-worn tags associated with player *x* is therefore given by Equation (5),

(5) $$C_{x}=\left\{x+U_{x,1},\ldots ,x+U_{x,N_{T}}\right\}.$$

*Traffic model:* The global and local layers operate in the same radio channel. Global-layer ranging uses transmit power $P_{g}$, while local-layer ranging uses a lower transmit power $P_{l}$.

A global position update requires the globally tracked tag to range with $N_{A}$ anchors at update rate $f_{g}$. If each global ranging occupies an airtime $\tau_{g}$, the global-layer activity probability of a player is approximated as in Equation (6),

(6) $$p_{g}=\min\left(1,N_{A}f_{g}\tau_{g}\right).$$

For the local layer, pose estimation requires the body-worn tags to update their inter-tag distances. The number of required local ranging transactions per pose update satisfies $M_{l}\in \left[N_{T}-1,\;N_{T}\left(N_{T}-1\right)/2\right]$: the lower bound corresponds to a star topology from a single reference tag, and the upper bound to a full mesh among the $N_{T}$ tags. The analysis below uses the full-mesh upper bound given by Equation (7),

(7) $$M_{l}=\frac{N_{T}\left(N_{T}-1\right)}{2},$$

which is a conservative worst case for airtime demand. For a local update rate $f_{l}$ and local ranging airtime $\tau_{l}$, the local-layer activity load per cluster is given by Equation (8),

(8) $$p_{l}=\min\left(1,M_{l}f_{l}\tau_{l}\right).$$

The condition given by Equation (9),

(9) $$M_{l}f_{l}\tau_{l}<1$$

is therefore a basic airtime feasibility constraint for completing one local pose update within the available update period. For example, increasing $N_{T}$ from 4 to 8 increases $M_{l}$ from 6 to 28, i.e., by a factor of 28/6≈4.67, showing how quickly local airtime demand grows with pose granularity.

*Channel model:* Channel fading is assumed Rayleigh with independent unit-mean exponential gains h∼Exp(1). Signal attenuation at distance *r* follows the power-law model $l\left(r\right)=r^{-\alpha }$, with path-loss exponent α>2. These assumptions provide a tractable first-order interference model and are consistent with stochastic-geometry analyses of inter-body networks [15]. Body shadowing, posture-dependent attenuation, and time-varying multipath are not modeled explicitly; their impact is partly assessed through the sensitivity analyses in Section 5 and Section 7, while extensions to dynamic body-centric channels are discussed in Section 8. Thermal noise can be included, but the dense co-channel operation considered here is interference-limited; the main analysis is therefore expressed in terms of SIR.

*Interference model:* The total interference experienced by a receiver is decomposed into three components as in Equation (10),

(10) $$I=I_{g}+I_{\mathrm{intra}}+I_{\mathrm{inter}},$$

where $I_{g}$ denotes interference from active global-layer transmissions, $I_{\mathrm{intra}}$ denotes local-layer interference generated by other tags within the same player cluster, and $I_{\mathrm{inter}}$ denotes local-layer interference generated by tags belonging to other player clusters.

The global interference is given by Equation (11),

(11) $$I_{g}=\sum_{x\in \Phi_{p}}B_{x,g}P_{g}h_{x,g}l(∥x+U_{x,g}∥),$$

where $B_{x,g}∼\mathrm{Bernoulli}\left(p_{g}\right)$ indicates whether the global tag of player *x* is active, and $U_{x,g}$ denotes the displacement of the globally tracked tag relative to the player center.

For a typical local receiver in the cluster centered at the origin, the intra-cluster local interference is given by Equation (12),

(12) $$I_{\mathrm{intra}}=\sum_{u\in C_{0}\setminus \left\{u_{0},u_{r}\right\}}B_{u,l}P_{l}h_{u}l(∥u-u_{r}∥),$$

where $u_{0}$ and $u_{r}$ denote the desired local transmitter and receiver, respectively, and $B_{u,l}$ indicates whether another local tag in the same cluster is simultaneously active. If local ranging within a player is deterministically scheduled, then $B_{u,l}=0$ for all $u\in C_{0}\setminus \left\{u_{0},u_{r}\right\}$, and $I_{\mathrm{intra}}=0$.

Finally, the inter-cluster local interference is given by Equation (13),

(13) $$I_{\mathrm{inter}}=\sum_{x\in \Phi_{p}\setminus \left\{0\right\}}\sum_{u\in C_{x}}B_{x,u,l}P_{l}h_{x,u}l(∥x+u-u_{r}∥),$$

where $B_{x,u,l}$ denotes the activity of local tag *u* in cluster *x*.

This decomposition separates the main physical interference mechanisms of the two-layer architecture: high-power global ranging, local interference within the same player, and local interference from nearby players.

**Remark** **1.** *Global- and local-layer ranging of the same player are time-multiplexed on the same UWB transceiver and therefore cannot overlap. No intra-cluster cross-layer interference term is thus required; cross-layer interference between different players is already captured by $I_{g}$ and $I_{\mathrm{inter}}$.*

### 4.1. Ranging Success Probability

We evaluate performance from the perspective of a typical receiver under the Palm distribution of the corresponding point process [13]. A ranging transaction is considered successful if the received SIR exceeds a threshold θ. For a desired transmitter located at distance *r* from the receiver, the instantaneous SIR is given by Equation (14),

(14) $$\mathrm{SIR}=\frac{P_{t}h_{0}r^{-\alpha }}{I},$$

where $P_{t}\in \left\{P_{g},P_{l}\right\}$ depending on whether the considered link belongs to the global or local layer.

Under Rayleigh fading, the ranging success probability is given by Equation (15),

(15) $$p_{s}\left(r\right)=P\left(\mathrm{SIR}>\theta \right)=L_{I}\left(\frac{\theta r^{\alpha }}{P_{t}}\right),$$

where $L_{I}\left(s\right)=E\left[\exp\left(-sI\right)\right]$ is the Laplace transform of the aggregate interference.

In this work, a ranging operation is modeled as a single airtime-consuming transaction of duration $\tau_{g}$ or $\tau_{l}$. Therefore, unlike a packet-level model of double-sided two-way ranging that explicitly evaluates the success of each individual message, the success probability in (15) directly represents the success probability of a ranging transaction. The duration of the transaction enters the model through the activity probabilities $p_{g}$ and $p_{l}$ in (6) and (8).

Remark: A DS-TWR transaction requires successful POLL, RESP, and FINAL frames, so an exact treatment involves the joint event $\mathrm{Pr}({\mathrm{SIR}}_{1}>\theta ,{\mathrm{SIR}}_{2}>\theta ,{\mathrm{SIR}}_{3}>\theta )$. Only under independent equal-probability frame events would this reduce to $p^{3}$; in practice, repeated transmissions experience temporally correlated interference [16,17]. For tractability, we therefore model the complete DS-TWR exchange as a single airtime-consuming SIR event. This transaction-level approximation targets system-level interference and scalability trends rather than packet-level failure probabilities. Its limitation is reflected experimentally in Section 6, where the interference range exceeds the range for reliable three-frame DS-TWR.

For local ranging, the shorter airtime is achieved by using a shorter preamble configuration. This reduces channel occupancy but may also reduce the baseline ranging reliability, as packet synchronization becomes harder. We account for this effect through an empirical reliability factor $\gamma (\tau_{l})\leq 1$, obtained from Table 3, such that the local ranging success probability is given by Equation (16),

(16) $$p_{s,l}\left(r,\tau_{l}\right)=\gamma \left(\tau_{l}\right)L_{I_{g}}\left(s_{l}\right)L_{I_{\mathrm{intra}}}\left(s_{l}\right)L_{I_{\mathrm{inter}}}\left(s_{l}\right),\;s_{l}=\frac{\theta r^{\alpha }}{P_{l}}.$$

For the reference, longest-preamble configuration $\tau_{l,0}=1024\;\mathrm{sym}$, we set $\gamma (\tau_{l,0})=1$.

Similarly, for global ranging, the success probability is given by Equation (17),

(17) $$p_{s,g}\left(r\right)=L_{I_{g}}\left(s_{g}\right)L_{I_{\mathrm{inter}}}\left(s_{g}\right),\;s_{g}=\frac{\theta r^{\alpha }}{P_{g}},$$

where local-layer transmissions from all wearable clusters contribute to $I_{\mathrm{inter}}$. Intra-cluster interference is not defined for the global receiver when the receiver is an anchor.

### 4.2. Laplace Transforms of the Interference Components

The global tags form an independently thinned version of the parent process, with activity probability $p_{g}$. Approximating the displacement of the global tag from the player center as negligible compared with global anchor distances, the global interference field is a PPP with density $p_{g}\lambda_{p}$. Its Laplace transform is the standard PPP expression [13]

(18) $$L_{I_{g}}\left(s\right)=\exp\left(-2\pi \lambda_{p}p_{g}\int_{0}^{\infty }\left(1-\frac{1}{1+sP_{g}r^{-\alpha }}\right)r\;dr\right).$$

For inter-cluster local interference, each player cluster contributes a finite number of possible local interferers. Assuming independent local activity with probability $p_{l,t}$ per tag, the Laplace transform is

(19) $$L_{I_{\mathrm{inter}}}\left(s\right)=\exp\left(-2\pi \lambda_{p}\int_{0}^{\infty }\left[1-G_{l}\left(s,v\right)\right]v\;dv\right),$$

where

(20) $$G_{l}\left(s,v\right)={\left(1-p_{l,t}+p_{l,t}\int_{R^{2}}\frac{1}{1+sP_{l}{∥v+u∥}^{-\alpha }}f_{U}\left(u\right)\;du\right)}^{N_{T}}.$$

In Equation (20), *v* denotes the distance from the interfering player center to the typical receiver, and the exponent $N_{T}$ appears because each cluster contains a deterministic number of tags. The per-tag local activity probability $p_{l,t}$ is in turn related to the cluster-level local load through Equation (21):

(21) $$p_{l,t}\approx \frac{2p_{l}}{N_{T}}.$$

Each pairwise DS-TWR transaction involves two tags, and both endpoints transmit during different parts of the three-frame exchange. Thus, averaging the transaction load over the $N_{T}$ tags gives the factor $2p_{l}/N_{T}$. This convention is used throughout the numerical results in Section 5 and Section 6.

For the intra-cluster interference term in the uncoordinated local case, conditioned on the relative positions of the tags in the typical cluster, the Laplace transform is

(22) $$L_{I_{\mathrm{intra}}}\left(s\right)=\prod_{u\in C_{0}\setminus \left\{u_{0},u_{r}\right\}}\left(1-p_{l,t}+\frac{p_{l,t}}{1+sP_{l}{∥u-u_{r}∥}^{-\alpha }}\right).$$

For scheduled local ranging within the player, intra-cluster interference is eliminated and

(23) $$L_{I_{\mathrm{intra}}}\left(s\right)=1.$$

## 5. Interference Analysis and Scaling Limits

Scalability is evaluated in terms of the ranging success probability obtained from the model parameters $N_{P}$, $N_{T}$, $N_{A}$, $f_{g}$, $f_{l}$, $\tau_{g}$, $\tau_{l}$, $P_{g}$, and $P_{l}$, which combine sport-dependent deployment choices with the hardware operating points characterized in Section 3.

The maximum supported player density for a target ranging reliability $p_{0}$ is defined as

(24) $$\lambda_{p}^{\max}=\sup\left\{\lambda_{p}:p_{s}\left(r;\lambda_{p},N_{T},N_{A},f_{g},f_{l},\tau_{g},\tau_{l}\right)\geq p_{0}\right\}.$$

From Equation (24), the corresponding maximum number of players in a finite field follows as

(25) $$N_{P}^{\max}=\left\lfloor \lambda_{p}^{\max}A_{\mathrm{field}}\right\rfloor .$$

Together, Equations (24) and (25) translate the reliability target $p_{0}$ into an admissible deployment size. Similarly, for a fixed player density, the maximum local update rate follows from Equation (26):

(26) $$f_{l}^{\max}=\sup\left\{f_{l}:p_{s,l}\left(r;\lambda_{p},N_{T},f_{l},\tau_{l}\right)\geq p_{0}\right\},$$

subject to the airtime feasibility constraint in (9). Table 5 summarizes the reference parameter values used in the numerical results of Section 5. The ranging airtimes, transmit-power settings, and empirical reliability factor are based on the DW3110 operating points characterized in Section 3, while the remaining parameters define the reference propagation and spatial-model assumptions.

### 5.1. Local Layer Analysis

For local-layer performance, we consider a typical player cluster and condition on one desired transmitter–receiver pair within that cluster. The desired local ranging distance is denoted by $r_{l}$. The local success probability is given by (16).

Two local access modes are considered. In the uncoordinated mode, multiple body-worn tags on the same player may initiate ranging transactions independently, giving rise to intra-cluster interference according to Equation (22).

(27) $$p_{s,l}^{\mathrm{sched}}\left(r_{l},\tau_{l}\right)=\gamma \left(\tau_{l}\right)L_{I_{g}}\left(s_{l}\right)L_{I_{\mathrm{inter}}}\left(s_{l}\right).$$

Table 6 is computed from Equation (16) using Equations (18) and (19), together with either Equation (22) (ALOHA) or Equation (23) (coordinated).

**Figure 4:**
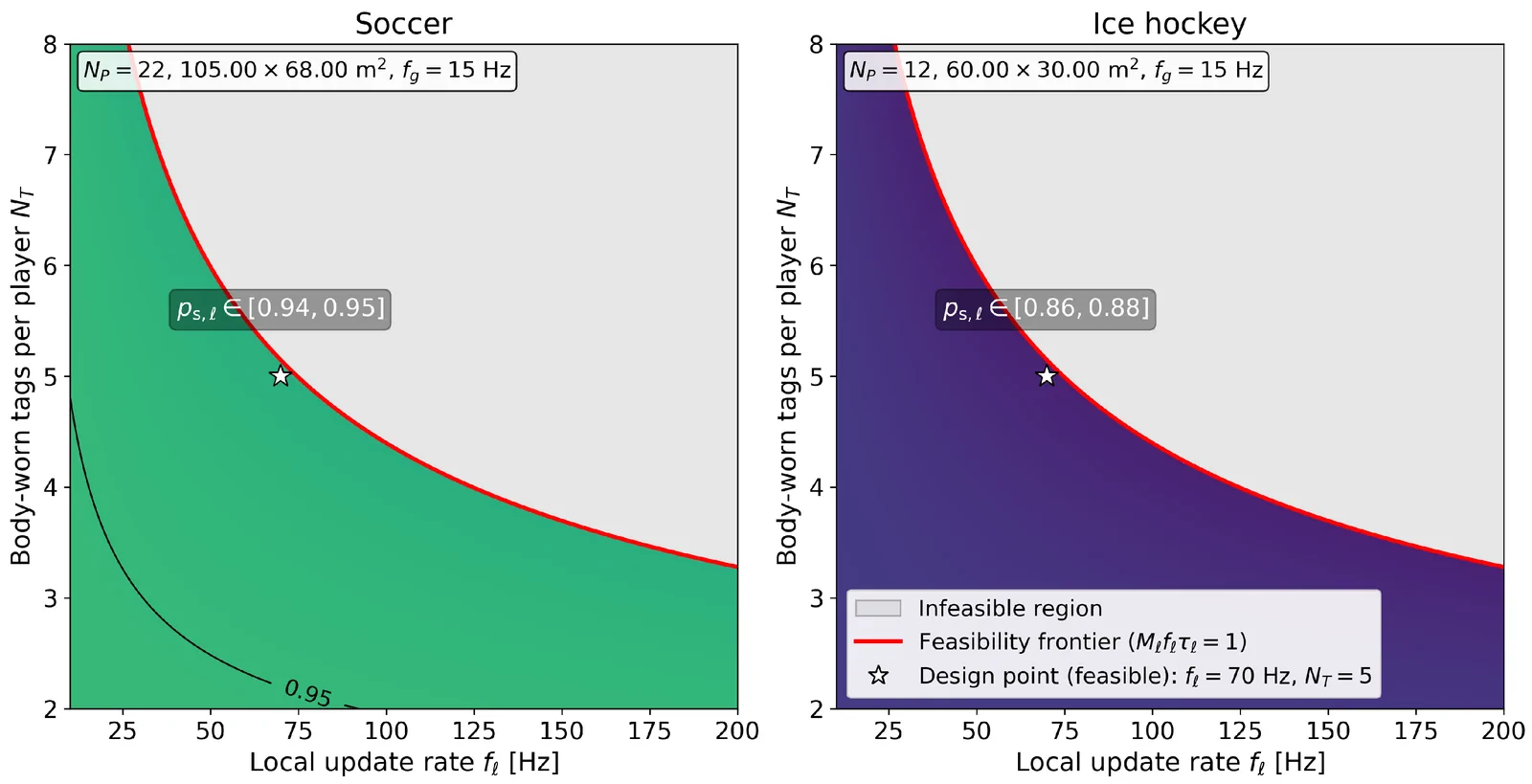
Local-layer success probability $p_{s,l}$ over local update rate $f_{l}$ and tags per player $N_{T}$, with coordinated intra-cluster access. Left: soccer ($N_{P}=22$, 105×68 m^2^). Right: ice hockey ($N_{P}=12$, 60×30 m^2^). The red curve is the airtime feasibility frontier from (9); gray shading marks infeasible configurations. Stars mark nominal design points. Because $I_{g}$ is constant in $\left(f_{l},N_{T}\right)$ and the inter-cluster load is bounded by $\lambda_{p}$, reliability varies only marginally within the feasible region.

Figure 4 evaluates (16) over $\left(f_{l},N_{T}\right)$ for two representative sports from Table 1: soccer ($N_{P}=22$ on a 105×68 m^2^ field) and ice hockey ($N_{P}=12$ on a 60×30 m^2^ rink), with coordinated intra-cluster access. The red frontier marks $M_{l}f_{l}\tau_{l}=1$, separating airtime-feasible designs from infeasible ones. Within the feasible region, $p_{s,l}$ varies only marginally (soccer: ≈0.95; ice hockey: ≈0.86–0.88). This flatness arises because global interference $I_{g}$ does not depend on $\left(f_{l},N_{T}\right)$ at fixed $N_{P}$ and $f_{g}$, while inter-cluster local interference scales with $\lambda_{p}\left(M_{l}f_{l}\tau_{l}\right)$ and is therefore capped by the player density $\lambda_{p}$ inside the feasible set. The dominant $\left(f_{l},N_{T}\right)$ tradeoff exposed by the figure is therefore whether a full local pose update can be completed within one period, rather than large reliability gradients across feasible configurations. We do not include a volleyball panel in this figure because the much higher player density shifts most of the $\left(f_{l},N_{T}\right)$ sweep into low-reliability regimes; volleyball is analyzed explicitly in Section 6 with focus on proximity-aware coordination.

**Figure 5:**
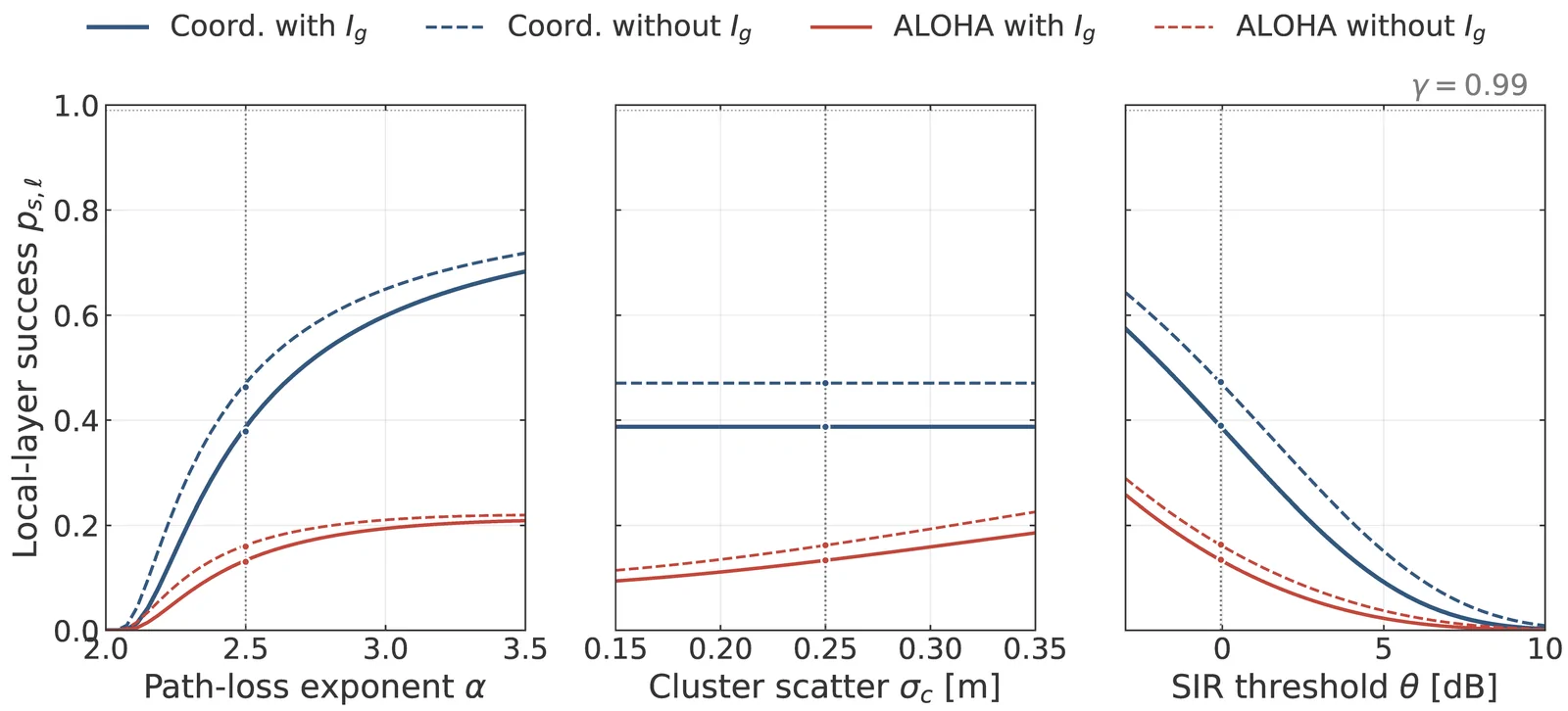
Sensitivity of the local-layer success probability $p_{s,l}$ to α, $\sigma_{c}$, and θ for the volleyball operating point ($\lambda_{P}=0.074$ m^−2^, $N_{A}=20$). Curves correspond to the four cases of Table 6; vertical dotted lines mark the reference values and the horizontal line γ=0.99.

Figure 5 assesses the sensitivity of the local-layer results to the channel and cluster parameters α, $\sigma_{c}$, and θ by repeating the four volleyball cases of Table 6 over the indicated ranges. One parameter is varied at a time, with the others fixed at the reference values of Table 5. Volleyball is used as the highest-density and most interference-limited scenario.

The three sweeps reveal different sensitivities of the local layer. The dependence on α is mainly driven by inter-player interference: a larger α suppresses contributions from spatially separated transmitters more rapidly, so the resulting reduction in inter-player interference is most pronounced in the dense volleyball scenario. In contrast, $\sigma_{c}$ primarily affects the geometry of interferers within a player cluster. It therefore changes the ALOHA cases, where several nearby body-worn tags may overlap in time, but becomes irrelevant once intra-player transmissions are coordinated and $I_{\mathrm{intra}}$ is removed. The SIR threshold θ acts differently, since it changes the decoding requirement itself and therefore shifts all operating cases simultaneously. The figure shows that the absolute success probabilities depend on the propagation, cluster geometry, and decoding assumptions, but that intra-player coordination remains the dominant local-layer scalability mechanism. The empirical factor γ is not swept because it is measurement-derived and enters $p_{s,l}$ multiplicatively.

The spatial player model is kept fixed in this analysis. Robustness to finite-field effects and non-Poisson player distributions is therefore not assessed here and is discussed as a limitation in Section 8.

### 5.2. Global Layer Analysis

For global-layer performance, we consider a typical global ranging link between a player tag and an infrastructure anchor at distance $r_{g}$. The desired transmitter uses power $P_{g}$, while interference is generated by active global tags and local-layer transmissions occurring simultaneously in the wearable network. The global success probability then takes the form of Equation (28):

(28) $$p_{s,g}\left(r_{g}\right)=L_{I_{g}}\left(s_{g}\right)L_{I_{\mathrm{inter}}}\left(s_{g}\right),\;s_{g}=\frac{\theta r_{g}^{\alpha }}{P_{g}}.$$

**Figure 6:**
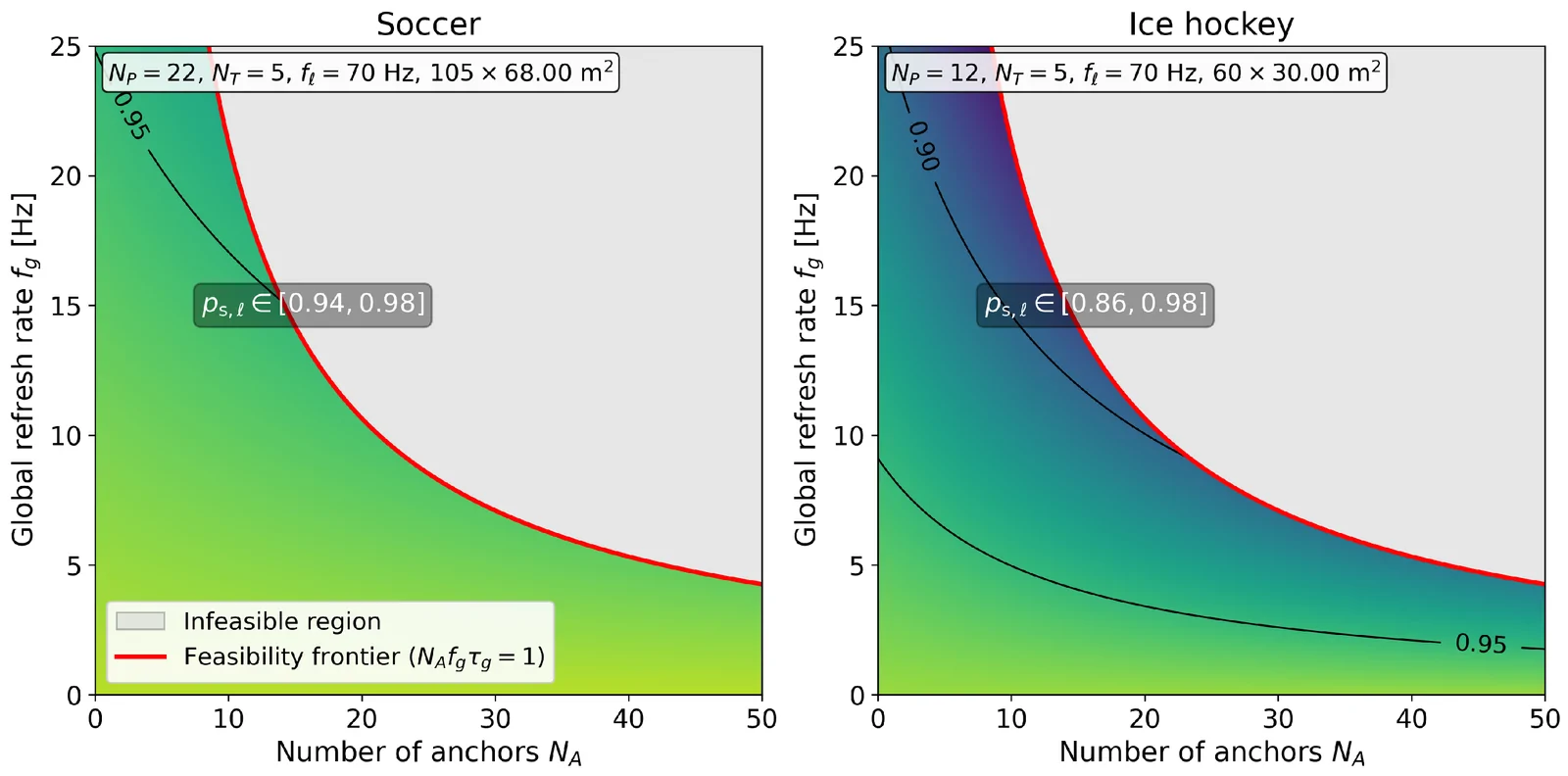
Local-layer success probability $p_{s,l}$ versus anchors $N_{A}$ and global refresh rate $f_{g}$, with coordinated intra-cluster access and fixed $N_{T}=5$, $f_{l}=70$ Hz. Left: soccer ($N_{P}=22$). Right: ice hockey ($N_{P}=12$). The red curve is the airtime feasibility frontier $N_{A}f_{g}\tau_{g}=1$ from (6); gray shading marks infeasible configurations. Annotated ranges report $p_{s,l}$ over the feasible region.

Increasing $N_{A}$ or $f_{g}$ increases the global-layer activity probability $p_{g}$ in (6), and therefore increases the global interference term. Increasing $N_{T}$ or $f_{l}$ increases the local-layer activity load in (8), and therefore increases the local interference seen by both global and local ranging links. Figure 6 evaluates (16) over $\left(N_{A},f_{g}\right)$ for soccer and ice hockey, with coordinated intra-cluster access, fixed $N_{T}=5$, and $f_{l}=70$ Hz. The red frontier marks $N_{A}f_{g}\tau_{g}=1$, separating feasible global designs from infeasible ones. Within the feasible region, $p_{s,l}$ decreases as either $N_{A}$ or $f_{g}$ increases (soccer: ≈0.94–0.98; ice hockey: ≈0.87–0.98), reflecting the stronger global interference load at higher anchor counts and refresh rates. The dominant $\left(N_{A},f_{g}\right)$ tradeoff is therefore twofold: first, whether a full global position update can be completed within one period, and second, how much global-layer activity the chosen parameters inject into the shared channel.

## 6. Scaling via Proximity-Aware Local Coordination

The previous section quantified scalability limits under increasing player density, update rates, and body-worn sensor counts. Architectural separation between global and local ranging already reduces interference, but dense sport scenarios still require additional coexistence mechanisms.

One standard option is to exploit PHY diversity, for example, by distributing local ranging transactions across quasi-orthogonal preamble codes. If $N_{\mathrm{PC}}$ preamble codes are available and transactions are uniformly assigned to them, the effective density of simultaneous local interferers is approximately thinned as $\lambda_{\mathrm{eff}}\approx \lambda_{P}/N_{\mathrm{PC}}$. This approach can reduce aggregate interference and can be combined with the method proposed here. However, because PHY diversity is a standard interference-thinning mechanism and is constrained in practice by code availability, synchronization robustness, and receiver complexity, it is not analyzed further.

Our approach is to use the approximate player positions already provided by the global localization layer to drive local medium-access coordination. The key idea is to introduce temporal separation only where it is physically needed: between players that are close enough for their local ranging transactions to create strong mutual interference. The pairwise inter-player distance between players *i* and *j* is defined in Equation (29):

(29) $$d_{ij}=∥x_{i}-x_{j}∥.$$

These distances are therefore available without adding a separate sensing or discovery mechanism.

Instead of enforcing complete network-wide TDMA scheduling, local coordination is activated only when players become closer than a predefined proximity threshold $d_{\mathrm{th}}$. Players fulfilling the proximity criterion in Equation (30)

(30) $$d_{ij}<d_{\mathrm{th}}$$

are temporarily assigned to different local coordination slots, so that the strongest local interferers are separated in time. Players outside such congestion regions remain uncoordinated and continue to reuse the same local ranging resources. This avoids the main drawback of network-wide TDMA: fixed temporal separation among all players, including pairs that are already sufficiently separated in space and therefore do not need coordination.

If $N_{S}$ coordination slots are used within a local congestion region, the effective local refresh rate for the coordinated players is defined by Equation (31):

(31) $$f_{l,\mathrm{eff}}=\frac{f_{l}}{N_{S}}.$$

Thus, increasing $N_{S}$ reduces the probability of harmful temporal overlap within a congestion region and improves ranging reliability, but it also lowers the effective pose refresh rate of the players participating in that local coordination group. The scalability advantage comes from applying this rate penalty only locally, instead of imposing it on the complete deployment.

To quantify this tradeoff, we use the bounded-cluster coordination model expressed in Equation (32), built on the same Laplace-form success expression used in the previous section:

(32) $$p_{s,l}=\gamma \;L_{\mathrm{intra}}\;L_{\mathrm{inter}}\;L_{\mathrm{global}}.$$

To obtain a tractable approximation relating the slot count to residual inter-player interference, consider a dense congestion component of *K* player networks. Its uncoordinated active-tag intensity is $\lambda_{P}N_{T}f_{l}\tau_{l}$. With $N_{S}$ coordination slots, we approximate the fraction of player networks that remain temporally overlapping as

(33) $$q_{\mathrm{res}}\left(N_{S},K\right)=\max\;\left(1-\frac{N_{S}}{K},0\right).$$

**Figure 7:**
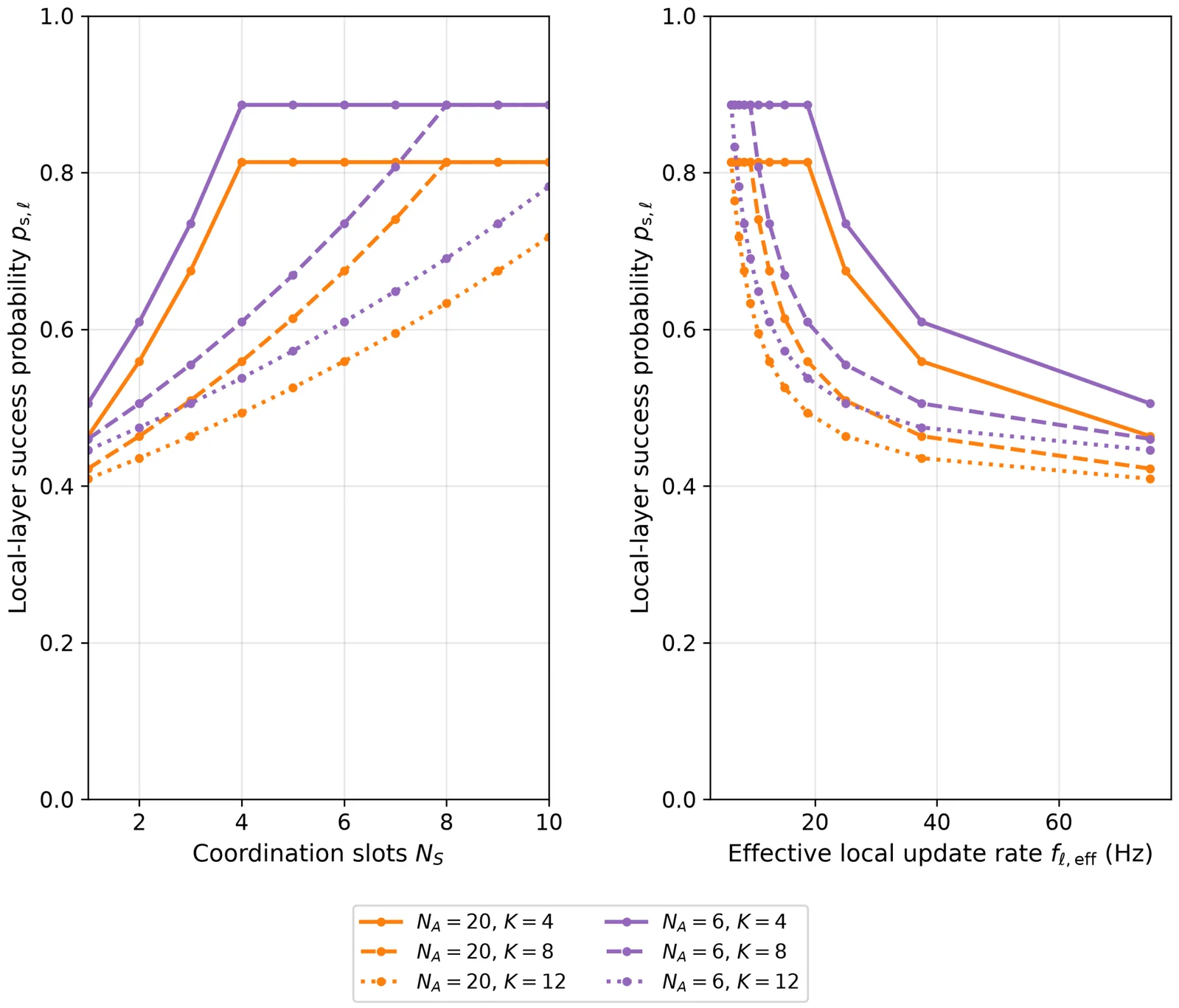
Proximity-aware coordination tradeoff for volleyball. Left: local-layer success probability versus coordination slots $N_{S}\in \left\{1,\ldots ,12\right\}$. Right: local-layer success probability versus effective local update rate $f_{l,\mathrm{eff}}=f_{l}/N_{S}$. Colors denote $N_{A}\in \left\{20,6\right\}$; line styles denote K∈{4,8,12} (solid/dashed/dotted). Slotting improves reliability but reduces effective temporal resolution.

Thinning the uncoordinated activity by this factor gives

(34) $$\lambda_{l}^{\left(\mathrm{coord}\right)}\left(N_{S},K\right)=\lambda_{P}N_{T}f_{l}\tau_{l}\max\;\left(1-\frac{N_{S}}{K},0\right),$$

which is the partial-coordination model used in Figure 7. For $N_{S}<K$, players without an orthogonal slot retain ALOHA-like access and generate residual interference; for $N_{S}\geq K$, the component can be fully separated. The residual-interference model captured by Equations (33) and (34) is a heuristic approximation most representative of dense, near-complete congestion graphs. In a general proximity graph, nonadjacent players may reuse the same slot, so the fraction of vertices left unassigned by DSATUR does not generally equal $(K-N_{S})/K$.

Algorithm 1 implements the slot assignment using greedy DSATUR coloring with at most $N_{S}^{\max}$ colors. If additional colors are required, the unassigned players remain uncoordinated, consistent with the residual-interference model above. Alternatively, overflow players could defer ranging to a later round, eliminating residual interference at the cost of additional latency and reduced update rate.

Figure 7 evaluates the reliability–rate tradeoff of proximity-aware coordination for volleyball, the highest-density scenario considered in this work. We consider local congestion clusters of size K∈{4,8,12} and two anchor deployments, $N_{A}\in \left\{20,6\right\}$. The left panel shows how the local-layer success probability changes with the number of coordination slots $N_{S}$, while the right panel expresses the same operating points in terms of the effective local update rate $f_{l,\mathrm{eff}}=f_{l}/N_{S}$.

Without inter-player coordination ($N_{S}=1$), the local-layer success probability is only about 0.42–0.50, depending on *K* and $N_{A}$. For a four-player congestion cluster, using $N_{S}=4$ slots raises $p_{s,l}$ to approximately 0.81 for $N_{A}=20$ and 0.89 for $N_{A}=6$, corresponding to an absolute gain of about 34–39 percentage points, or a relative improvement of roughly 1.7–1.8 times. This is obtained while retaining an effective local update rate of $f_{l,\mathrm{eff}}=75/4=18.75$ Hz for the coordinated players.

Larger congestion clusters require more slots to reach the same reliability. For K=8, $N_{S}=8$ restores the same high-reliability regime, with $p_{s,l}\approx 0.81$–0.89, but the effective local update rate decreases to 9.4 Hz, below the target range in Table 1. For K=12, the evaluated range up to $N_{S}=10$ still improves reliability to approximately 0.72–0.78, but does not fully reach the four- and eight-player plateau. Thus, proximity-aware coordination provides a scalable compromise: it applies TDMA-like temporal separation only inside local congestion regions, where the reliability gain is largest, while spatially separated players continue to reuse the same local ranging resources without paying the penalty of a network-wide TDMA.
**Algorithm 1** Proximity-aware local coordination. The global layer provides approximate player positions, which are used to identify nearby players requiring temporal separation.**Require:** Player positions ${\left\{x_{i}\right\}}_{i=1}^{N_{P}}$ from the global layer, threshold $d_{\mathrm{th}}$, maximum number of slots $N_{S}^{\max}$**Ensure:** Slot assignment $s_{i}$ for local ranging 1:Initialize all players as uncoordinated: $s_{i}\leftarrow 0$ 2:Build proximity graph G=(V,E) with one vertex per player 3:**for** each pair of players (i,j) **do** 4:    **if** $∥x_{i}-x_{j}∥<d_{\mathrm{th}}$ **then** 5:        Add edge (i,j) to E 6:    **end if** 7:**end for** 8:**for** each connected component C of G **do** 9:    **if** |C|=1 **then**10:        Keep player uncoordinated11:    **else**12:        Compute greedy DSATUR coloring of C with palette $\left\{1,\ldots ,N_{S}^{\max}\right\}$13:        **if** DSATUR requires more than $N_{S}^{\max}$ colors **then**14:           Unassigned vertices remain uncoordinated15:        **end if**16:    **end if**17:**end for**18:Players with $s_{i}=0$ operate without slot restriction19:Players with $s_{i}>0$ perform local ranging only in their assigned slot

**Remark on signaling and synchronization:** Proximity-aware coordination reuses information and signaling already available in the localization system. The proximity graph can be updated at the global-layer cadence $f_{g}$ (approximately every 40–100 ms in Table 1), and the slot assignment $s_{i}\in \left\{0,\ldots ,N_{S}^{\max}\right\}$ can be included in the control broadcast for the subsequent local round, requiring only $\left\lceil \log_{2}\left(N_{S}^{\max}+1\right)\right\rceil$ bits per player in addition to protocol headers. The same broadcast provides the timing reference, so no separate synchronization exchange is required. Coordination latency is therefore on the order of one global-layer update interval, while the required guard time is characterized experimentally in Section 6.2.

**Remark on effective update rate:** The reduced rates in Figure 7 apply only while players belong to a congestion region. For example, K=4 and K=8 give $f_{l,\mathrm{eff}}=18.75$ Hz and 9.4 Hz, respectively, substantially below the 50–100 Hz target range of Table 1. This temporarily reduces the temporal resolution available for pose tracking, but must be weighed against uncoordinated operation, which in the same regime yields below 50% ranging success in Figure 7 and consequently a large fraction of missing range measurements. Once the players separate, they return to the nominal rate. The practical impact of this local and transient reliability–resolution tradeoff therefore depends on the frequency, duration, and size of congestion events in the specific sport application.

### 6.1. Comparison with Alternative MAC Schemes

Table 7 compares uncoordinated ALOHA, network-wide TDMA, and proximity-aware coordination for an illustrative volleyball configuration with $N_{P}=12$ and one K=4 congestion group, corresponding to the case in Figure 7. The remaining eight players are assumed sufficiently separated for spatial reuse. ALOHA preserves the nominal 75 Hz rate but gives only $p_{s,l}=0.45$ inside the congestion group; network-wide TDMA restores near-isolated reliability at 6.25 Hz for every player. Proximity-aware coordination restricts the rate penalty to the four nearby players, which operate at 18.75 Hz while the remaining players retain 75 Hz.

The comparison exposes the intended reliability–rate tradeoff: ALOHA maximizes temporal resolution but becomes unreliable under local contention, whereas network-wide TDMA restores reliability by penalizing the complete deployment. Proximity-aware coordination exploits spatial reuse to confine this penalty to the congestion region. This tradeoff is consistent with prior UWB scalability studies. Ridolfi et al. [6] analytically compare ALOHA with scheduled TDMA and hybrid access schemes; distributed TDMA [7], dense swarm-ranging protocols [18], and centralized ranging schedulers [8] provide more sophisticated realizations of coordinated medium access for dense UWB networks. Across these approaches, stronger temporal coordination improves reliability at the cost of update rate, scheduling overhead, or reduced spatial reuse.

### 6.2. Experimental Validation of Proximity-Aware Coordination

The purpose of the following experiments is to test whether the main insights of our analytical framework also appear in a small, finite-node implementation with real DS-TWR timing, receiver behavior, and protocol abort conditions. In particular, we evaluate whether a lightweight coordination action is enough to restore reliable ranging for two nearby players, each equipped with an initiator and a responder node.

The global layer is represented by a LEADER node that periodically broadcasts the start of the pose update routine to both players via a TRIGGER message. The two players have already been identified as belonging to the coordinated cluster. The experiment does not implement the full proximity-graph construction of Algorithm 1; instead, it validates the corresponding coordination action. Extension to larger components and mixed coordinated/uncoordinated populations is left to future work.

**Figure 8:**
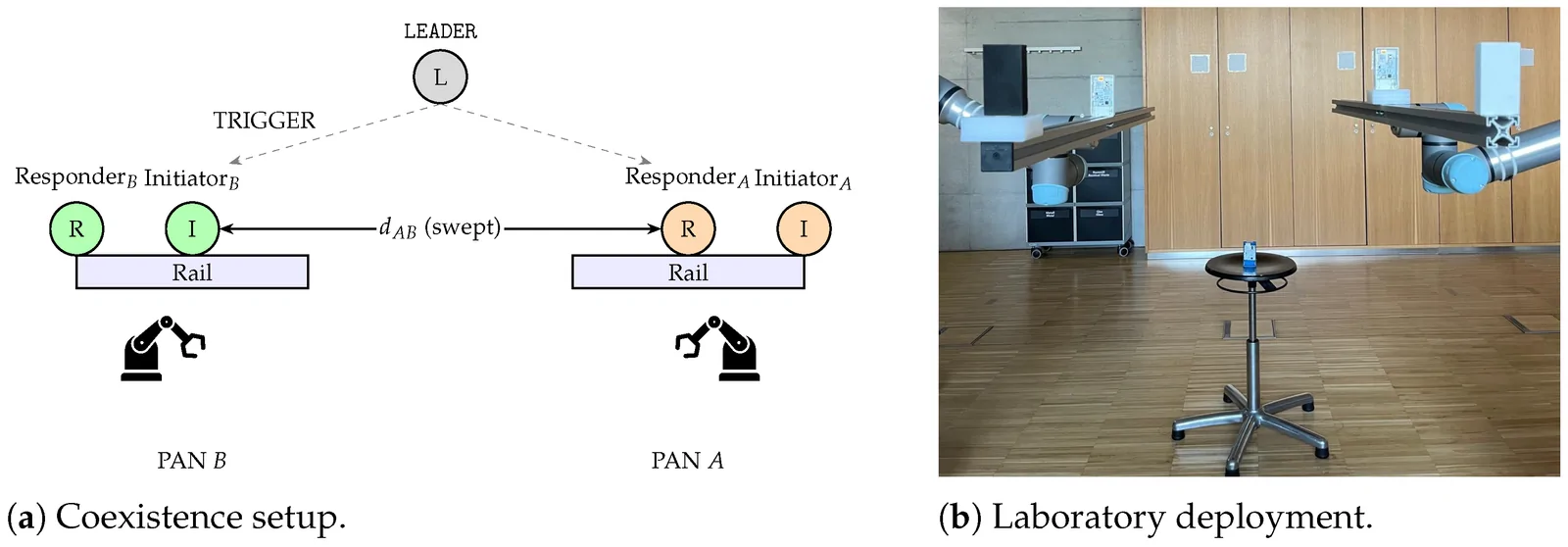
Two-network coexistence. The LEADER broadcasts the TRIGGER consumed by PAN (A and B). The inter-network distance $d_{\mathrm{AB}}$ (i.e., the gap between the two innermost cross-PAN nodes) is varied via the available cobots.

The two-network setup shown in Figure 8 isolates the distance-dependent coexistence problem that motivates proximity-aware coordination. The LEADER at the center of the setup broadcasts TRIGGER messages consumed by both PANs A and B. PAN A occupies the right rail: its initiator is placed at the outer edge of the rail and its responder at the rail midpoint. PAN B occupies the left rail in a mirror-flipped arrangement, with its initiator at the rail midpoint and its responder at the outer edge. The inter-network distance $d_{\mathrm{AB}}$ is defined as the gap between the two innermost cross-PAN nodes, namely the responder of PAN A and the initiator of PAN B. This distance is swept by translating both rails via the cobots. When the $d_{\mathrm{AB}}$ is small, the players are in the congestion region and temporal separation should be coordinated. When the $d_{\mathrm{AB}}$ is sufficiently large, the two networks can operate independently. Results are averaged over 5000 DS-TWR rounds per operating point.

We compare an uncoordinated baseline, in which the players operate independently regardless of their separation, with a coordinated regime in which the proximity-aware coordination is implemented as a temporal delay between the start of the pose update routine of the second player (PAN B) at each round, with the first player (PAN A) starting its round immediately after the TRIGGER. The delay for PAN B is drawn independently in each round from a clipped Gaussian distribution, emulating lightweight pairwise coordination with timing uncertainty rather than perfectly synchronized network-wide TDMA. Two settings are tested: a tight-guard configuration with delays drawn from a clipped Gaussian with a mean of 2.0 ms and a clipping interval [1.4,3.0] ms, and a wider-guard configuration that raises the lower clip to [1.6,3.0] ms with the same mean. Both use σ=0.05 ms and fit within the 45 ms round period.

The delay windows are chosen relative to the measured duration of a 32-symbol preamble DS-TWR round. Thus, the tight-guard setting (μ=2.0, [1.4,3.0]) leaves only a narrow margin above the round duration, whereas the wider-guard setting (μ=2.0, [1.6,3.0]) provides additional tolerance for implementation-level timing uncertainty. In the uncoordinated regime, $d_{\mathrm{AB}}$ is swept from 40 cm to 140 cm to identify the distance beyond which explicit coordination is not necessary.

Table 8 reports the resulting success rates. For each round, the firmware classifies the DS-TWR outcome on each PAN as **ok**, **intf**, or **to**. An **ok** outcome means that the full DS-TWR exchange was completed. An **intf** outcome means that a frame with a mismatched PAN identifier was decoded inside an expected receive window. A **to** outcome means that no decodable frame was received. The success rate reported in Table 8 is the fraction of **ok** outcomes across all attempts.

Table 8 provides a key validation of the coordination principle. If reliability is defined as a DS-TWR success probability above 90%, the uncoordinated networks are not jointly reliable until $d_{\mathrm{AB}}=140$ cm. At 40 cm, the two networks almost completely collapse, with success of only 2% and 8%. At 80 cm, PAN A reaches 89%, but PAN B remains at only 21%, so ranging remains unreliable. Even at 120 cm, PAN B reaches only 72%. In contrast, with the more relaxed coordinated delay, both players already reach a 99% success rate at $d_{\mathrm{AB}}=40$ cm. Thus, the experiment shows that temporal separation is essential in close proximity, and that a lightweight pairwise delay is sufficient to restore near-isolated ranging performance.

The coordinated rows demonstrate the practical effect of the proposed mechanism. The tight coordination already improves the 40 cm case from 2%/8% to 97%/84%, confirming that separating the two local rounds in time removes most cross-PAN interference. The configuration with the larger guard interval (last row) reaches 99% for both PANs. This confirms that the local delay must include sufficient guard time for implementation-level timing uncertainty. This requirement is compatible with the analytical coordination model: the model captures the benefit of separating nearby local transmissions, while the experiment identifies the timing margin needed to realize this separation on the hardware platform.

**Remark on ranging and interference ranges:** The single-network measurements of Table 3 show that DS-TWR at $P_{l}$ with a 32-symbol preamble is reliable only up to approximately 60 cm, and already degrades strongly between 60 and 80 cm. However, Table 8 and Table 9 show that another local network can still cause interference at larger separations. The asymmetric degradation remains visible up to 120 cm. This is consistent with the fact that a successful DS-TWR exchange requires three consecutive frames to be received correctly and within their timing windows, whereas interference involves the reception or partial reception of a single frame.

**Remark on asymmetry between players:** The strong asymmetry between PAN A and PAN B in the uncoordinated regime is not related to our analytical model, but to the specific ranging-update implementation used in the experiment. To maintain high update rates, the firmware uses tight receive windows and aborts a ranging attempt when the expected POLL is not received, or when a frame from the wrong PAN is received. Because PAN A starts slightly earlier, PAN B may receive PAN A’s POLL during its receive window and consequently abort its own ranging attempt. PAN A can complete the ranging more often, while PAN B accumulates more **intf** and **to** outcomes. This asymmetry explains the intermediate-distance behavior of the table without changing the main conclusion: uncoordinated operation is not reliable when the local networks are within mutual interference range.

Overall, our hardware validation confirms that the stochastic-geometry insights remain valid in a finite-node implementation.

### 6.3. Two-Network Coexistence: Analytical Model vs. Measurements

To compare the transaction-level SIR abstraction with the finite-node experiment, we specialize (16) to one desired local link and one deterministic co-channel interferer at distance $d_{\mathrm{AB}}$, neglecting all other players and global-layer interference. This gives

(35) $$p_{\mathrm{pred}}\left(d_{\mathrm{AB}}\right)=\frac{\gamma }{1+\theta {\left(r_{l}/d_{\mathrm{AB}}\right)}^{\alpha }}.$$

Table 10 compares this prediction with the uncoordinated measurements of Table 8 and reports the residuals for both PANs.

The model captures the transition from strong interference toward spatial reuse, but not the asymmetric recovery of the two PANs. Residuals range from only 3 percentage points for PAN A at 60 cm to 40 percentage points for PAN B at 80 cm, where firmware-level receive-window and abort behavior dominate. The distance dependence is also sharper in hardware than under continuous power-law/Rayleigh propagation: Table 9 shows single-frame success falling from 98% at 80 cm to zero at 160 cm. Receiver sensitivity, packet detection, timing windows, and abort logic are therefore important implementation effects outside the transaction-level SIR abstraction.

Accordingly, the analytical model is used to capture system-level interference and spatial-reuse trends, while the measurements determine the practical coexistence limits of the implemented DS-TWR system. The comparison in Table 10 should therefore be interpreted qualitatively rather than as a quantitative calibration. The SIR model captures the transition from strong mutual interference toward spatial reuse, whereas the success probabilities at individual operating points can differ substantially because implementation effects such as receive-window timing and firmware-abort behavior are not represented. The predicted $p_{s,l}$ values are therefore used primarily to identify interference trends and operating regimes rather than as calibrated per-configuration reliability estimates.

### 6.4. Proof-of-Concept Test with Two Five-Sensor Players

**Figure 9:**
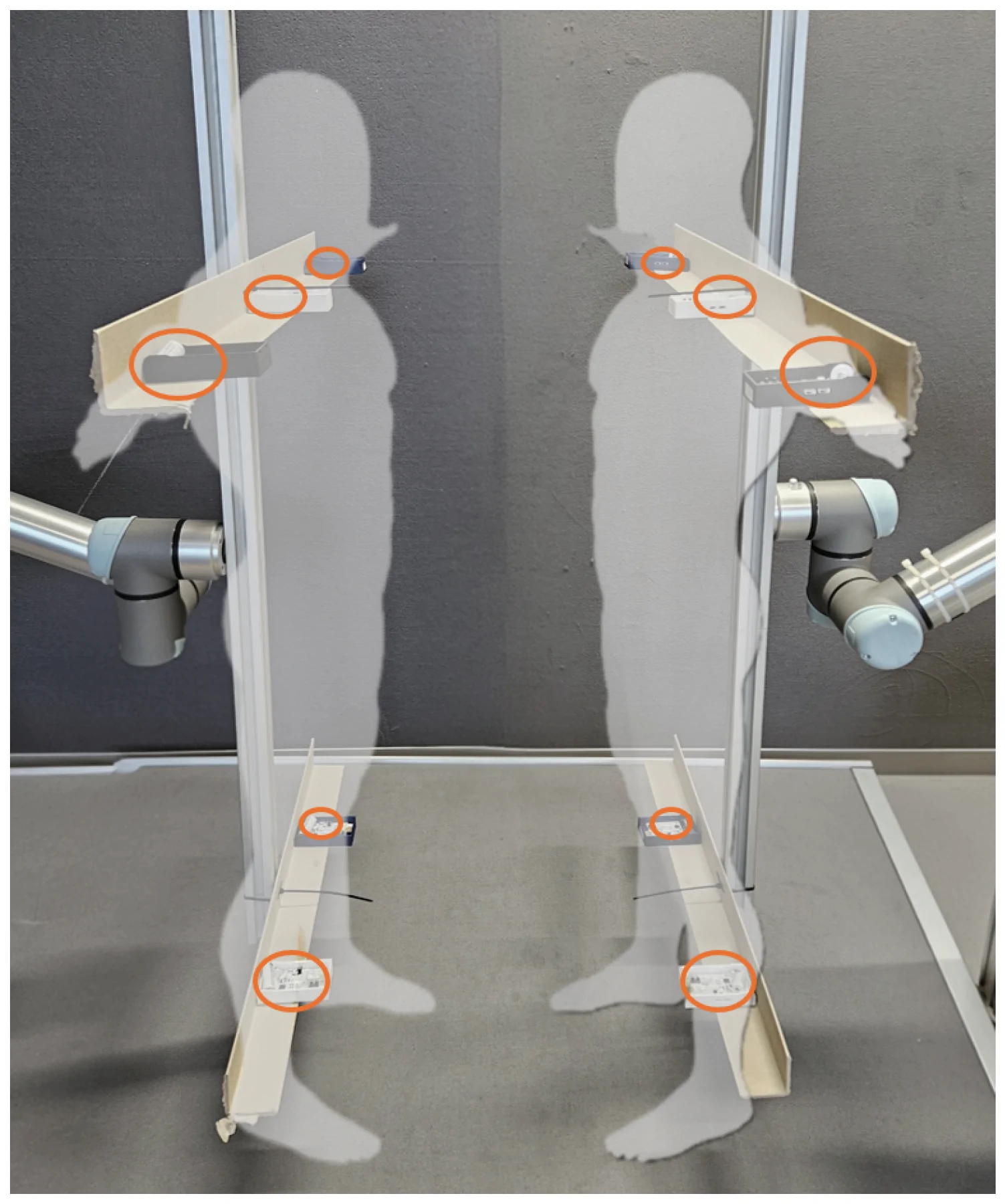
Proof-of-concept setup with two players and five sensors per player. Each player carries one INITIATOR (torso) and four RESPONDERs (wrists and ankles), highlighted by the orange circles. The two silhouettes are separated by $d_{\mathrm{AB}}=40$ cm.

This final experiment populates each player network with five body-worn sensors, so that every ranging round contains a sequence of intra-PAN DS-TWR exchanges. Each player is equipped with five sensors, as shown in Figure 9, so that links span the actual body-scale distances. The two players are again placed at $d_{\mathrm{AB}}=40$ cm, the worst-case proximity condition, and the coordination setup is otherwise unchanged.

The proof-of-concept uses a full-mesh ranging topology among the five body-worn sensors of each player. Consequently, every pose-update round contains $M_{l}=\frac{N_{T}\left(N_{T}-1\right)}{2}=10$ pairwise DS-TWR exchanges per player. The nominal ranging airtime of the ten exchanges is approximately $10\tau_{l}\approx 13.4$ ms, corresponding to an airtime-limited rate of approximately 75 Hz if implementation overhead were neglected. On the tested platform, however, the complete sequence requires approximately 45 ms once inter-exchange gaps and firmware-level processing are included, limiting the demonstrated PoC rate to approximately 22 Hz. The inter-PAN delay of PAN B is therefore increased accordingly, using a clipped Gaussian distribution with μ=50 ms and σ=1.5 ms, clamped to [48,55] ms, so that PAN B starts after the local ranging sequence of PAN A is expected to have completed.

Table 11 presents the experiment outcome breakdown. PAN A and B reach a success rate of 99.92% and 99.90%, respectively. Interference-labeled outcomes (**intf**) remain in the single digits on both PANs, showing that the coordinated inter-PAN delay effectively suppresses cross-PAN frame decoding inside expected receive windows even when ten pairwise intra-PAN exchanges are chained per round. The proof-of-concept confirms that the proposed lightweight coordination scales to a realistic five-sensor-per-player full-mesh configuration. Aggregated over all ten pairwise intra-PAN exchanges, both networks retain near-isolated ranging performance at the worst-case proximity distance of 40 cm, without any explicit intra-PAN scheduling beyond the fixed intra-round order.

The proof-of-concept therefore demonstrates reliable coordination for a realistic five-sensor-per-player full-mesh configuration at the implemented round timing, rather than full rate scalability to the targets of Table 1. The gap between the 13.4 ms nominal ranging airtime and the 45 ms implemented sequence indicates substantial timing headroom, but achieving the target rates requires firmware and scheduling optimization that is not evaluated here.

## 7. Sensitivity Analysis and Coordination Calibration

The reference parameters of Table 5 are used throughout Section 5 and Section 6. We next test the robustness of the proposed coordination mechanism to these assumptions and examine the calibration of its proximity threshold.

### 7.1. Sensitivity of the Proximity-Aware Coordination

**Figure 10:**
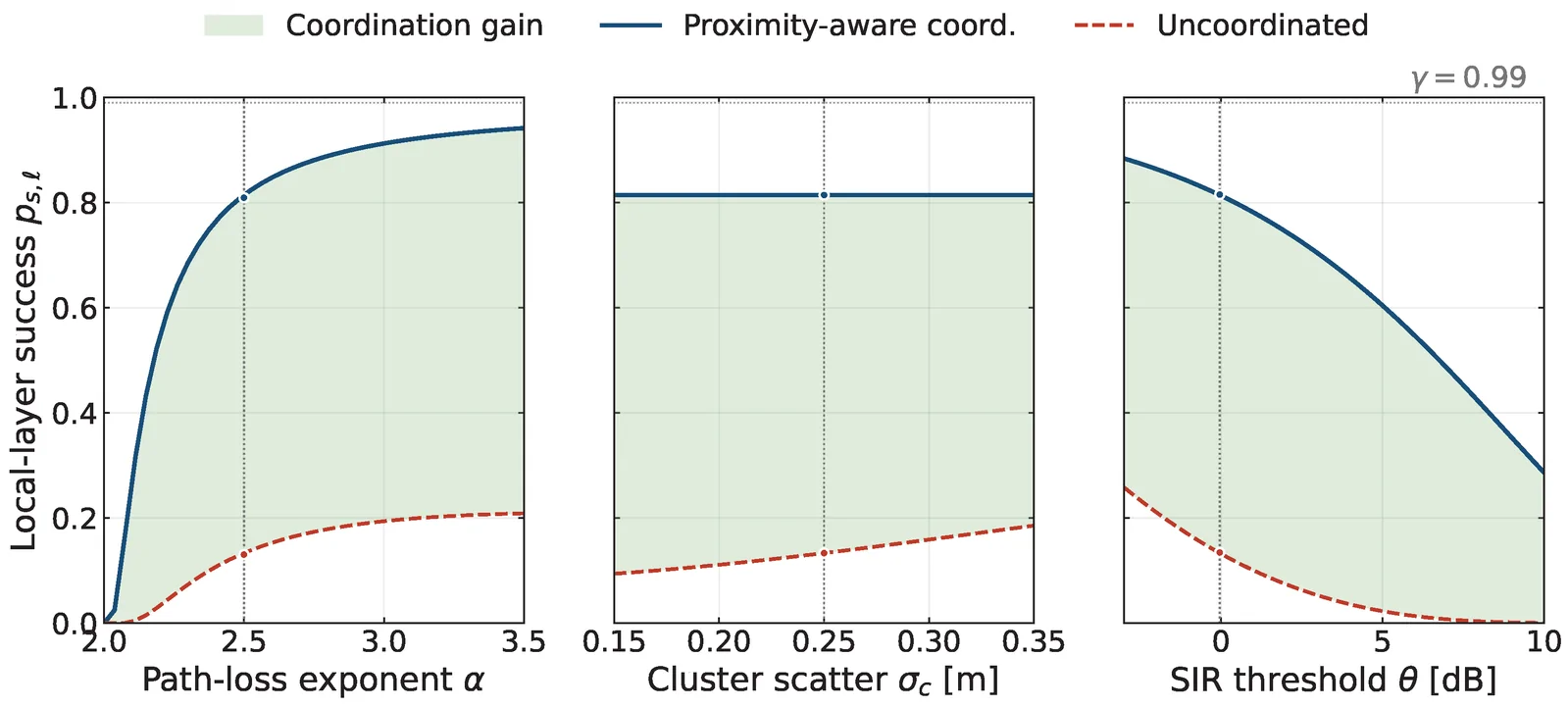
Sensitivity of $p_{s,l}$ under proximity-aware coordination to α, $\sigma_{c}$, and θ for the volleyball operating point. Parameter ranges and reference values are as in Figure 5, with $N_{S}=K=4$. The blue solid line shows the proximity-aware coordinated case ($N_{S}=K=4$), the red dashed line shows the uncoordinated ALOHA baseline, and the green shaded area highlights the operating range in which the coordinated case reaches the target reliability.

Figure 10 repeats the sensitivity sweep of Figure 5 for the proximity-aware scheme, using the same volleyball operating point and parameter ranges. A four-player congestion component with $N_{S}=K=4$ is compared with uncoordinated operation.

The trends are consistent with the earlier local-layer analysis, but proximity coordination removes the dominant nearby inter-player interference and therefore raises reliability substantially over most of the parameter range. At the reference point, $p_{s,l}$ increases from about 0.13 to 0.82. The coordinated case is also insensitive to $\sigma_{c}$, since separating nearby player networks into slots removes their interference. Although the absolute reliability remains sensitive to α and θ, the coordination gain persists across the considered ranges, showing that the result is not tied to the reference parameter set.

As in Figure 5, the spatial player model is kept fixed, so this sensitivity analysis does not assess robustness to finite-field or non-Poisson player distributions.

### 7.2. Calibration and Sensitivity of the Proximity Threshold

The threshold $d_{\mathrm{th}}$ should reflect the interference range rather than the isolated ranging range. At the selected local operating point, isolated DS-TWR already degrades beyond approximately 60–80 cm, whereas the coexistence measurements show that inter-player interference remains relevant through the 1.2–1.4 m transition region: the less reliable PAN reaches 21%, 72%, and 96% success at 0.8, 1.2, and 1.4 m, respectively.

We use $d_{\mathrm{th}}=1.4$ m as the reference operating point, consistent with the 90% reliability criterion adopted in Section 6.2. Table 12 quantifies the associated reliability–reuse tradeoff through the measured reuse reliability and the coordination area $A_{\mathrm{coord}}=\pi d_{\mathrm{th}}^{2}$. Reducing the threshold from 1.4 to 1.2 m decreases the potential coordination area to approximately 73% of the reference value, but also lowers the measured reuse reliability from 96% to 72%. Further reducing it to 0.8 m cuts the coordination area to approximately 33% of the reference value while leaving reuse reliability at only 21%.

$A_{\mathrm{coord}}$ is used only as a geometry-based indicator of potential coordination overhead; the actual group size and slot demand depend on the instantaneous player configuration. The selected 1.4 m threshold therefore prioritizes the target reliability, while 1.2 m represents a more aggressive spatial-reuse operating point that reduces coordination overhead at the cost of lower uncoordinated reliability.

## 8. Discussion of Results

The results of this paper support a common design message: dense wearable UWB motion capture should not be treated as a single flat ranging network. Field-level player localization and on-body pose tracking operate at different spatial scales, require different update rates, and create different interference footprints. Separating them into global and local layers therefore changes the medium-access problem. The global layer is responsible for field-level observability, while the local layer carries the high-rate body-scale ranging load. This architectural separation makes spatial reuse meaningful: local ranging resources can be reused by players that are sufficiently separated, while only nearby players require additional coordination.

A second consequence is that the main scalability bottleneck is not the number of players alone, but the airtime load created by the desired pose resolution. At the local layer, the number of pairwise ranging transactions grows quadratically with the number of body-worn tags. The model therefore exposes a direct tradeoff between tag count, local update rate, and ranging airtime. This explains why sparse and moderate-density sports leave a larger design margin, whereas dense or fast-motion scenarios move the system closer to its reliability boundary. In this sense, the stochastic-geometry model is not only a predictor of success probability, but also a design tool for selecting feasible combinations of pose granularity and refresh rate.

The results also suggest a hierarchical coordination strategy. Coordination within each player cluster is a platform-level requirement: the body-worn tags of one player should not contend independently if high local update rates are desired. This part of the design is largely independent of the sport scenario and can be implemented as a fixed local schedule. Coordination between players is different. It should depend on the current spatial configuration of the game, because only nearby players create strong local-layer interference. The proximity-aware strategy therefore avoids the main limitation of network-wide TDMA by applying a rate penalty only to players within local congestion regions.

This point also clarifies the relation between the analytical and experimental parts of the paper. The stochastic-geometry framework describes average scaling behavior across sport-dependent densities and system parameters. The experiments are qualitatively consistent with the physical mechanisms upon which the model relies: the existence of distance-dependent spatial reuse, the collapse of simultaneous local ranging in close proximity, and the recovery obtained by temporal separation. They further show that the practical coordination threshold should be tied to the interference/reuse distance of the implemented DS-TWR system, not only to the nominal ranging distance of an isolated link.

Compared with conventional UWB scalability approaches based on flat scheduling, distributed TDMA, or PHY-level interference thinning, the proposed architecture exploits information that is already available in the localization system. The global layer supplies approximate player positions, and these positions are reused to decide where local-layer coordination is necessary. This provides a spatial decision layer above existing MAC or PHY mechanisms. Preamble-code diversity, channelization, or backbone-assisted scheduling can still be used as complementary tools, but the central result here is that coordination need not be applied uniformly across the full deployment.

The present treatment remains limited in both modeling and experimental scope. The channel model does not explicitly capture body attenuation, player motion, time-varying multipath, or the dynamic formation of congestion regions. Experiments are restricted to controlled static LOS conditions, with proximity-aware coordination validated only for two nearby local networks. Extending the framework to dynamic body-centric channels, moving athletes, larger coordinated components, mixed coordinated/uncoordinated populations, and full in-game conditions remains for future work. The reduced effective update rate inside coordinated congestion regions also limits the temporal resolution available for pose tracking; its impact on specific sport motions will depend on the duration and size of such congestion events and remains to be evaluated experimentally.

The player process is modeled as an infinite homogeneous PPP for analytical tractability and cross-sport comparability. This neglects finite-field boundary effects, which generally reduce interference near the field limits [13,19], and the non-Poisson structure of real player formations. In particular, repulsive models such as Matérn hard-core processes can represent minimum inter-player spacing and suppress very close configurations admitted by a PPP [20]. These effects mainly alter the absolute interference distribution rather than the coordination principle studied here; finite-field and non-Poisson extensions are therefore left for future work.

## 9. Related Work

Prior work relevant to this manuscript can be grouped into three threads: (i) UWB tracking in sports and human motion analysis, (ii) UWB scalability and MAC/scheduling design, and (iii) stochastic-geometry modeling of dense interference-limited networks.

### 9.1. UWB Localization in Sports and Human Motion Analysis

The authors of [1,2] validate UWB player tracking in team-sport settings (handball, football, tennis) against optical or GPS references. Reference [3] demonstrates field-level player tracking in ice hockey using a commercial local positioning system. The authors of [21] compare UWB and GPS positioning side-by-side for tennis player tracking and identify residual limitations of UWB under those conditions, and [22] presents a deployment-oriented workflow for UWB anchor self-localization in large multi-hop deployments. Further, [4] demonstrates sub-meter body-worn UWB localization by explicitly modeling human-body shadowing, and [5] combines sparse inertial sensors with UWB inter-sensor ranging for scalable full-body motion capture, directly motivating the on-body pose layer studied here.

More recent work has extended these results. The authors of [23] characterize the ranging performance and multi-user interference robustness of the updated IEEE 802.15.4z standard, providing an important link between protocol specification and measured behavior in realistic environments. Finally, [24] studies end-to-end UWB TDOA deployment for mobile agents and shows how sensor placement and system-level design interact.

These works establish feasibility and accuracy, but typically do not provide sport-aware scalability rules under dense coexistence.

### 9.2. UWB Scalability, MAC, Scheduling, and Multi-User Ranging

References [6,7,8] show that MAC and scheduling dominate large-scale performance. Their results motivate explicit coexistence design, but mostly treat single-layer localization networks. Reference [9] demonstrates a hardware platform with a sub-GHz backbone link that partially offloads MAC overhead from the UWB channel, a concept that is orthogonal but complementary to the two-layer separation proposed here.

Addressing the multi-user ranging problem more directly, [18] proposes a UWB swarm-ranging protocol for dynamic and dense networks that achieves simultaneous ranging and communication at high node densities. The authors of [7] develop a fully distributed TDMA algorithm for mobile UWB networks. Compared with that work, the proximity-aware strategy studied here avoids network-wide TDMA by activating slot coordination only among nearby players, reducing overhead in sparse to moderate regimes. In addition, [6] provides theoretical scalability models for UWB localization across MAC and PHY configurations that inform the update-rate bounds used in our model.

For the specific problem of anchor-dense deployments, [25] addresses large-scale multi-zone TDOA localization by combining cascaded wireless multi-hop clock synchronization with dynamic localization-zone selection, demonstrating continuous cross-room coverage in a multi-room testbed. Finally, [26] proposes an adaptive channel-aware DL-TDOA scheme that dynamically adjusts ranging frequency based on non-line-of-sight conditions, reducing power consumption while maintaining accuracy.

These works largely treat homogeneous single-layer networks. The gap addressed by the present paper is the lack of a unified treatment that separates two qualitatively different ranging tasks and derives access rules from their different spatial and temporal scales.

### 9.3. Stochastic Geometry for Interference and Reliability

Foundational stochastic geometry tools and methodology are given by [13] and the tutorial of [27], while [14] analyzes interference and outage in clustered ad hoc networks using the Thomas cluster process, providing the Laplace-transform machinery that directly underlies our inter-cluster interference decomposition in Section 5. The authors of [28] extend this approach to heterogeneous networks in which users and small-cell base stations are co-clustered around shared hotspot centers via a Poisson cluster process, showing that within-cluster resource reuse improves throughput at the cost of coverage—a tradeoff analogous to the densely co-located wearable tags considered here.

For body-centric settings, [15,29,30] apply related ideas to WBAN interference analysis, and the recent survey by [31] reviews interference-mitigation strategies in WBANs spanning classical signal-processing, cooperative, game-theoretic, and learning-based approaches. The inter-WBAN model of [15] in particular uses an extended Matérn point process to capture MAC-induced spatial correlations, a modeling choice we revisit and adapt with the Thomas cluster process for wearable tags.

The SIR meta distribution, introduced by [32] and extended by [33] to per-link reliability and rate control, and by [34] to Poisson-cluster-process-based heterogeneous network models, provides a finer-grained performance view than the mean success probability used here. While the present paper focuses on the mean success probability as a tractable design metric, the meta-distribution framework would be a natural extension for characterizing the tail of link reliability.

Relative to these strands, the gap addressed in this paper is the lack of a unified, sport-aware framework that links (a) a two-layer localization architecture, (b) stochastic-geometry-based design interpretation across sport regimes, and (c) controlled coexistence experiments validating temporal-overlap and distance-dependent reuse mechanisms.

## 10. Conclusions and Future Work

This paper studied the scalability of dense wearable UWB motion capture using a two-layer architecture that separates field-level player localization from local on-body pose ranging. The main result is that this separation turns the medium-access problem into a spatial-reuse problem. A stochastic-geometry model was used to connect sport-dependent parameters, body-worn tag count, update rates, ranging airtime, and transmit power to reliability tradeoffs. Controlled DS-TWR experiments are qualitatively consistent with the key coexistence mechanisms behind the model: local ranging can be spatially reused when players are sufficiently separated, while nearby local networks require temporal coordination. Together, the analytical and experimental results show that proximity-aware coordination is a lightweight alternative to full network-wide synchronization, because it applies coordination only where player clustering makes it necessary.

Future work should extend the hardware validation to larger and dynamic player groups, including mixed coordinated and uncoordinated operation under in-game conditions. The analytical framework should likewise be extended to capture body attenuation, motion-induced NLOS and multipath, localization accuracy, and finite-field or non-Poisson player distributions. A further step is to replace the empirical reliability factor with a measurement-based function of distance, body placement, and PHY configuration, enabling more direct deployment-level design.

## Figures and Tables

**Table 1:** Deployment characteristics for soccer, volleyball, and ice hockey. The number of body-worn sensors and local update rates are aligned with sparse-inertial motion capture [5]. Global update rates are consistent with commercial UWB deployments [1].

Parameter	Soccer	Volleyball	Ice hockey
Playing area	105×68 m^2^	18×9 m^2^	60×30 m^2^
Number of players, $N_{P}$	22	12	12
Player density, $\lambda_{P}$	Low	High	Moderate
Global tags per player	1	1	1
Body-worn sensors per player, $N_{T}$	4–8	4–8	4–8
Global update rate, $f_{g}$	10–20 Hz	20–25 Hz *	20–25 Hz
Local update rate, $f_{l}$	50–100 Hz	50–100 Hz	50–100 Hz
Main scalability bottleneck	Field-wide coverage	Dense spatial reuse	Fast skating dynamics

* Player-tracking ceiling for volleyball; the upper bound is included as an aspirational design target matched to commercial UWB ball-tracking systems (e.g., Kinexon xBall [10]).

**Table 2:** Core system parameters used throughout the two-layer architecture and analytical model.

Symbol	Meaning
$N_{P}$	Number of players in the field
$N_{T}$	Total number of body-worn tags per player, including one global tag
$N_{A}$	Number of infrastructure anchors
$f_{g},f_{l}$	Global and local update rates
$\tau_{g},\tau_{l}$	Airtime per global/local ranging transaction
$P_{g},P_{l}$	Global/local transmit powers
$\lambda_{P}$	Player density, $N_{P}/A_{\mathrm{field}}$
$M_{l}$	Number of local pairwise ranges per pose update
$p_{g},p_{l}$	Global/local activity probabilities
$I_{g},I_{\mathrm{intra}},I_{\mathrm{inter}}$	Global, intra-cluster, and inter-cluster interference

**Table 3:** Short-range DS-TWR success probability as a function of preamble length, transmit-power setting, and ranging distance. Each entry is averaged over 5000 DS-TWR exchanges.

Preamble	TX Power	50 cm	60 cm	70 cm	80 cm	90 cm	100 cm
32 sym	$P_{l}$	98%	82%	33%	2%	0%	0%
1024 sym	$P_{l}$	99%	99%	99%	97%	26%	1%
32 sym	$P_{l}^{+}$	99%	99%	97%	90%	83%	68%

**Table 4:** Long-range DS-TWR success probability at elevated transmit power as a function of preamble length and inter-node distance. Each cell aggregates 5000 ranging exchanges.

Preamble	TX Power	1 m	5 m	10 m	15 m	20 m
32 sym	$P_{g}$	99%	99%	6%	0%	0%
1024 sym	$P_{g}$	99%	99%	99%	97%	3%

**Table 5:** Reference parameters used in the numerical evaluation.

Symbol	Value	Meaning/Basis
α	2.5	Reference path-loss exponent
θ	0.99 (≈0 dB)	Reference SIR decoding threshold
γ	0.99	Empirical reliability factor from Table 3 at $r_{l}=0.5$ m
$\sigma_{c}$	0.25 m	Reference body-worn tag-cluster spread
$r_{l}$	0.5 m	Reference local ranging distance
$P_{g}/P_{l}$	10 dB	Global-to-local power ratio
$\tau_{l}$	1339 μs	Measured local airtime, 32-symbol preamble
$\tau_{g}$	4695 μs	Measured global airtime, 1024-symbol preamble
$f_{U}\left(u\right)$	$N(0,\sigma_{c}^{2}I)$	iid Gaussian tag displacement in the Thomas cluster model

**Table 6:** Local-layer ranging success probability $p_{s,l}$ per sport. Deployment parameters: $N_{T}=6$, $f_{l}=75$ Hz, $f_{g}=10$ Hz, $N_{A}=20$. Channel path-loss α=2.5, SIR threshold θ=0.99, reliability factor γ=0.99, intra-player design distance $r_{l}=0.5$ m with $\sigma_{c}=0.25$ m, local airtime $\tau_{l}=1339\;\mu$s, global airtime $\tau_{g}=4695\;\mu$s. Intra-cluster coordination yields the dominant gain.

	Soccer	Volleyball	Ice Hockey
Player density $\lambda_{P}$ [m^−2^]	0.003	0.074	0.007
$p_{s,l}$ (Coordinated, with $I_{g}$)	0.954	0.387	0.910
$p_{s,l}$ (Coordinated, without $I_{g}$)	0.960	0.471	0.926
$p_{s,l}$ (ALOHA, with $I_{g}$)	0.329	0.133	0.313
$p_{s,l}$ (ALOHA, without $I_{g}$)	0.331	0.162	0.319

**Table 7:** Comparison of medium-access schemes for the illustrative volleyball configuration with $N_{P}=12$, $f_{l}=75$ Hz, and one K=4 congestion group. The reported $p_{s,l}$ refers to a player inside the congestion group.

Scheme	Cong.-Group Rate [Hz]	Sep.-Player Rate [Hz]	Avg. Rate $\bar{f_{\ell }}$ [Hz]	Cong.-Group $p_{s,\ell }$
Uncoordinated ALOHA	75.0	75.0	75.0	0.45
Network-wide TDMA	6.25	6.25	6.25	≈0.99
Proximity-aware (proposed)	18.75	75.0	56.25	≈0.81

**Table 8:** DS-TWR outcome breakdown for two nearby players. Uncoordinated baseline at $P_{l}$ from 40 cm to 140 cm and proximity-aware coordination at 40 cm. Each operating point aggregates 5000 rounds per PAN. **ok**: full DS-TWR exchange completed; **intf**: a frame with mismatched PAN; **to**: no decodable frame received. Success rate succ = **ok** / (**ok** + **intf** + **to**).

Interference Management	Distance	PAN A					PAN B			
		ok	intf	to	succ		ok	intf	to	succ
Uncoordinated	40 cm	132	14	4854	2%		408	98	4494	8%
Uncoordinated	60 cm	2327	659	2014	46%		773	2178	2049	15%
Uncoordinated	80 cm	4490	358	152	89%		1054	2514	1432	21%
Uncoordinated	120 cm	4918	47	35	98%		3609	614	777	72%
Uncoordinated	140 cm	4984	11	5	99%		4848	46	106	96%
Coord. (μ=2.0, [1.4,3.0])	40 cm	4866	12	122	97%		4245	5	750	84%
Coord. (μ=2.0, [1.6,3.0])	40 cm	4976	12	12	99%		4955	5	40	99%

**Table 9:** Single-frame success rate versus distance (operating point $P_{l}$ and 32-symbol preamble).

Distance	40 cm	60 cm	80 cm	100 cm	120 cm	140 cm	160 cm
Success rate	100%	100%	98%	77%	14%	13%	0%

**Table 10:** Simplified two-network SIR prediction versus the uncoordinated measurements of Table 8. Residuals are $p_{\mathrm{pred}}-p_{\mathrm{obs}}$.

$d_{\mathrm{AB}}$ [cm]	$p_{\mathrm{pred}}$	$p_{\mathrm{obs},A}$	$p_{\mathrm{obs},B}$	Res. A	Res. B	Measured Regime
40	0.22	0.03	0.08	+0.19	+0.14	Strong mutual interference
60	0.44	0.47	0.15	−0.03	+0.29	Transition
80	0.61	0.90	0.21	−0.29	+0.40	Asymmetric recovery
120	0.81	0.98	0.72	−0.17	+0.09	Partial spatial reuse
140	0.86	0.99	0.97	−0.13	−0.11	Reliable spatial reuse

**Table 11:** Proof-of-concept outcome breakdown. Inter-PAN delay (μ=50 ms, σ=1.5 ms, clamped to [48,55] ms) at $d_{\mathrm{AB}}=40$ cm. **to** aggregates POLL/RESP/FINAL timeouts and CRC errors.

PAN	Rounds	DS-TWR exch.	ok	intf	to	S
PAN A	2500	25 000	24 979	1	20	99.92%
PAN B	2500	25 000	24 976	5	19	99.90%

**Table 12:** Sensitivity of the proximity threshold. Measured reuse reliability is the lower success probability of the two uncoordinated local networks; $A_{\mathrm{coord}}=\pi d_{\mathrm{th}}^{2}$ indicates the potential coordination extent and is normalized to the 1.4 m reference.

$d_{\mathrm{th}}$ [m]	$A_{\mathrm{coord}}$ [m^2^]	Relative Coordination Area	Measured Reuse Reliability
0.8	2.01	0.33	0.21
1.2	4.52	0.73	0.72
1.4	6.16	1.00	0.96

## Data Availability

Data available on request due to restrictions (e.g., privacy, legal or ethical reasons).

## Abbreviations

The following abbreviations are used in this manuscript:

UWB	Ultra-Wideband
DS-TWR	Double-Sided Two-Way Ranging
PPP	Poisson Point Process
PHY	Physical Layer
MAC	Medium Access Control
TDMA	Time-Division Multiple Access
SIR	Signal-to-Interference Ratio
PAN	Personal Area Network
